# Phosphate limitation enhances malic acid production on nitrogen-rich molasses with *Ustilago trichophora*

**DOI:** 10.1186/s13068-024-02543-z

**Published:** 2024-07-03

**Authors:** Luca Antonia Grebe, Philipp Georg Lichtenberg, Katharina Hürter, Eva Forsten, Katharina Miebach, Jochen Büchs, Jørgen Barsett Magnus

**Affiliations:** AVT-Biochemical Engineering, RWTH Aachen University, Forckenbeckstraße 51, 52074 Aachen, Germany

**Keywords:** *Ustilago trichophora*, Malic acid, Platform chemical, Molasses, Alternative feedstock, Phosphate limitation, Internal phosphate storage, Bioavailability, Online monitoring, Bioeconomy

## Abstract

**Background:**

An important step in replacing petrochemical products with sustainable, cost-effective alternatives is the use of feedstocks other than, e.g., pure glucose in the fermentative production of platform chemicals. Ustilaginaceae offer the advantages of a wide substrate spectrum and naturally produce a versatile range of value-added compounds under nitrogen limitation. A promising candidate is the dicarboxylic acid malic acid, which may be applied as an acidulant in the food industry, a chelating agent in pharmaceuticals, or in biobased polymer production. However, fermentable residue streams from the food and agricultural industry with high nitrogen content, e.g., sugar beet molasses, are unsuited for processes with Ustilaginaceae, as they result in low product yields due to high biomass and low product formation.

**Results:**

This study uncovers challenges in evaluating complex feedstock applicability for microbial production processes, highlighting the role of secondary substrate limitations, internal storage molecules, and incomplete assimilation of these substrates. A microliter-scale screening method with online monitoring of microbial respiration was developed using malic acid production with *Ustilago trichophora* on molasses as an application example. Investigation into nitrogen, phosphate, sulphate, and magnesium limitations on a defined minimal medium demonstrated successful malic acid production under nitrogen and phosphate limitation. Furthermore, a reduction of nitrogen and phosphate in the elemental composition of *U. trichophora* was revealed under the respective secondary substrate limitation. These adaptive changes in combination with the intricate metabolic response hinder mathematical prediction of product formation and make the presented screening methodology for complex feedstocks imperative. In the next step, the screening was transferred to a molasses-based complex medium. It was determined that the organism assimilated only 25% and 50% of the elemental nitrogen and phosphorus present in molasses, respectively. Due to the overall low content of bioavailable phosphorus in molasses, the replacement of the state-of-the-art nitrogen limitation was shown to increase malic acid production by 65%.

**Conclusion:**

The identification of phosphate as a superior secondary substrate limitation for enhanced malic acid production opens up new opportunities for the effective utilization of molasses as a more sustainable and cost-effective substrate than, e.g., pure glucose for biobased platform chemical production.

**Supplementary Information:**

The online version contains supplementary material available at 10.1186/s13068-024-02543-z.

## Background

In the transition from a fossil-based to a sustainable bioeconomy, biotechnologically produced platform chemicals are of particular interest. These biomass-derived chemicals have multiple functional groups and can thus be converted into several high-value chemicals and materials [1]. This versatility makes them potential building blocks for a wide range of applications. However, the production cost for sustainable platform chemicals is high, compared to fossil-based bulk chemicals. As a result, they are currently mainly used in niche applications.

The C4 dicarboxylic acid malic acid was selected to be among the 12 most important platform chemicals. It has the potential to be a key building block for deriving both commodity and specialty chemicals [2]. Currently, malic acid is mainly used as an acidulant and taste enhancer in the food and beverage industry [3]. The dicarboxylic nature of malic acid also makes it a potential intermediate for polymer production similar to polylactic acid [4]. Future non-food applications of malic acid may include personal care, pharmaceuticals, metal cleaning and finishing, and textile finishing [4, 5]. Malic acid also has the potential to replace the fossil-based commodity chemical maleic anhydride [1]. In 2023, the malic acid market reached 222.3 million US$, and an annual growth of 4.4% is expected for the years 2024 to 2032 [6].

Malic acid is a key intermediate of the tricarboxylic acid (TCA) cycle and is thus ubiquitous in nature [7]. Nevertheless, it does not usually accumulate under normal growth conditions, but only in mutant cells or under specific stress conditions [4]. The first patent on malic acid production was already filed in 1960 identifying *Aspergillus* sp. as an efficient natural producer [8]. However, production processes with *Aspergillus* sp. have certain drawbacks, such as a high shear susceptibility of the filamentous organism and high sensitivity towards trace elements, making pretreatment of complex substrates crucial [9, 10]. The Ustilaginaceae, a family of smut fungi, are known to naturally produce a wide range of value-added chemicals under nitrogen limitation [11]. The wild-type strain *Ustilago trichophora* was found to naturally produce high amounts of malic acid, while offering the advantages of unicellular, yeast-like growth [12].

One of the major challenges of sustainable platform chemical production is the reduction of the overall fermentation costs [19]. Techno-economic analysis revealed that the main cost factor is linked to substrate costs [13]. Switching from pure sugar to fermentable residue streams not only lowers production costs, but also relieves competition with food production. Additionally, most residue streams from food and agricultural industry are currently only used for low-value applications and are hereby interesting substrates for value-added products [14]. An industrially relevant alternative feedstock is molasses, a by-product of sugar manufacturing. About 50 million tons of molasses are produced worldwide every year [3]. Currently, the majority is used as animal feed or as a substrate in ethanol or baker’s yeast production [15–17]. The regional availability and lower cost of sugar beet molasses render it an interesting residue stream for sustainable platform chemical production [3, 18]. Molasses contains large amounts of readily available sugars and can potentially be used in fermentations without any prior pretreatment [18]. However, the naturally occurring nitrogenous compounds, such as proteins, amino acids, and betaine, pose a challenge for processes requiring a specific carbon to nitrogen (C/N) ratio as with Ustilaginaceae [17, 19]. A common solution to overcome the unfavorable C/N ratio in molasses is the supplementation of purified sugar [20]. Although effective, this strategy collides with the initial motivation of using residue streams. A promising approach might be a switch to an alternative secondary substrate limitation. Opposed to 2.5 wt% nitrogen, contents of phosphate, sulphate, and magnesium in molasses from the Rhenish region are much lower with 0.02 wt%, < 0.6 wt%, and 0.005 wt%, respectively, making them potential optimization targets for secondary substrate limitation [18]. Research exploring alternatives to the state-of-the-art nitrogen limitation is typically conducted using defined minimal media. For other processes, alternative secondary substrate limitations have previously been identified: *Rhodosporidium toruloides* showed lipid accumulation, when exposed to phosphate or sulphate limitation on glucose [21, 22], *Aspergillus niger* exhibited citric acid production under phosphate- or iron-limited conditions using sucrose or glucose, respectively [23, 24], and *Yarrowia lipolytica* displayed high lipid accumulation under dual constraints of nitrogen and magnesium, using either glucose or glycerol as substrates [25]. Data on real residue streams, however, remain scarce.

This work bridges the gap between laboratory conditions and real-world residue streams. The aim is to provide a suitable screening method based on the model system of malic acid production with *U. trichophora* on molasses, and thereby expand the applicability of residue streams to produce biobased platform chemicals.

## Materials and methods

### Microorganism

All experiments were performed with the strain *Ustilago trichophora* TZ1, henceforth named *U. trichophora*, which was kindly provided by the Institute of Applied Microbiology of Prof. Lars Blank (RWTH Aachen University, Aachen, Germany) [12].

### Cultivation conditions and media

All shake-flask cultivations were performed in unbaffled 250 mL shake flasks with a filling volume (V_L_) of 20 mL, at a cultivation temperature (T) of 30 °C, a shaking frequency (n) of 350 rpm, and a shaking diameter (d_0_) of 50 mm (Climo-Shaker ISF1-X, Adolf Kühner AG, Birsfelden, Switzerland), if not specified otherwise. When using the adapted Verduyn medium for unlimited growth, the V_L_ was decreased to 8 mL to prevent oxygen limitation. All microtiter plate (MTP) cultivations were performed in 96 round U-bottom deep well plates with a well volume of 2 mL (VWR, Radnor (PA), United States). If not specified otherwise, V_L_ = 100 µL was used and cultivations were carried out at T = 30 °C, n = 1000 rpm, d_0_ = 3 mm, and a humidity of 85%. To maintain sterility, the MTPs were sealed with AeraSeal film BS-25 (Excel Scientific, Inc., Victorville (CA), United States).

Adapted versions of the Verduyn medium according to Geiser et al*.* were used for the experiments [26]. All chemicals were sourced from either Carl Roth GmbH + Co. KG (Karlsruhe, Germany), Merck KGaA (Darmstadt, Germany), or VWR International (Radnor, PA, United States). The preculture medium consisted of 50 g/L sucrose, 4 g/L NH_4_Cl, 2 g/L KH_2_PO_4_, 0.4 g/L MgSO_4_ • 7 H_2_O, 0.01 g/L FeSO_4_ • 7 H_2_O, 1 mL/L trace element solution and 0.1 M 2-(N-morpholino)ethanesulfonic acid (MES) buffer [27]. The trace element solution contained 15 g/L ethylenediaminetetraacetic acid, 4.5 g/L ZnSO_4_ • 7 H_2_O, 0.84 g/L MnCl_2_ • 2 H_2_O, 0.3 g/L CoCl_2_ • 6 H_2_O, 0.3 g/L CuSO_4_ • 5 H_2_O, 0.4 g/L Na_2_MoO_4_ • 2 H_2_O, 4.5 g/L CaCl_2_ • 2 H_2_O, 3 g/L FeSO_4_ • 7 H_2_O, 1 g/L H_3_BO_3_, 0.1 g/L KI. For main culture experiments investigating the buffer concentration and the influence of the carbon source, the medium consisted of 25 g/L sucrose, 0.8 g/L NH_4_Cl, 0.5 g/L KH_2_PO_4_, 0.2 g/L MgSO_4_ • 7 H_2_O, 0.01 g/L FeSO_4_ • 7 H_2_O, 1 mL/L trace element solution and 0.3 M MES buffer, if not specified otherwise. For experiments investigating the scale-down to MTPs and secondary substrate limitations, an adapted Verduyn medium for unlimited growth was developed, containing 25 g/L sucrose, 6 g/L NH_4_Cl, 2 g/L KH_2_PO_4_, 0.4 g/L MgSO_4_ • 7 H_2_O, 0.01 g/L FeSO_4_ • 7 H_2_O, 1 mL/L trace element solution and 0.3 M MES buffer. For each limitation step, the concentration of the respective salt was reduced and the respective counterion was supplemented equimolarly (Additional file 1: Table S1, Table S2). When using molasses as a carbon source, molasses was added until a sucrose concentration of 25 g/L was reached. The sucrose concentration of the molasses was determined by high performance liquid chromatography (HPLC) measurements. To facilitate handling, molasses was diluted with distilled water to 500 g molasses per liter stock solution before medium preparation. Stock solutions for sucrose, molasses, NH_4_Cl, KH_2_SO_4_, and MgSO_4_ were prepared separately and sterilized by autoclaving. The pH value of the KH_2_PO_4_ solution was adjusted to 6.5 with 10 M NaOH. Stock solutions for FeSO_4_, trace elements, and MES buffer were prepared separately and sterilized via sterile filtration. The pH value of MES buffer was adjusted to 7.2 with NaOH pellets. Stock solutions of sucrose, molasses, FeSO_4,_ and trace elements were stored at 4 °C. All other components were stored at room temperature.

Beet molasses was kindly provided by Pfeifer & Langen GmbH & Co. KG (Jülich, Germany). As Helm et al*.* analyzed a sample from the same manufacturer and site, the published elemental composition of 2.45 wt% nitrogen, 199 mg/kg phosphorus, < 0.6 wt% sulphur, and 50.5 mg/kg magnesium was used for the calculation of the respective carbon to secondary substrate (C/E) ratios of the medium [18]. Further data on the carbon and nitrogen sources contained in the molasses are also published in this work. Molasses stock solution contained 280 g/L sucrose equivalents based on cumulated sucrose, glucose, and fructose concentrations measured via HPLC. When molasses was diluted to a concentration of 25 g/L, 1.09 g/L elemental nitrogen, 0.0088 g/L phosphorus, 0.266 g/L sulphur, and 0.0022 g/L magnesium were hereby added to the cultivation medium. Both, secondary substrates derived from the molasses itself and from additional salts from the Verduyn medium, were considered in the calculation of the C/E ratio.

Precultures were inoculated from cryo cultures to an optical density (OD_600_) of 0.1 (details see in chapter offline sample analytics) and harvested during exponential growth. Main cultures were inoculated to an OD_600_ of 0.1. Precultures for the experiments investigating secondary substrate limitations were harvested and afterwards centrifuged with 1780 g at 4 °C for 10 min. The supernatant was discarded, and the cells were washed once with 9 g/L NaCl. The cells were resuspended in the respective medium of the main culture without the investigated secondary substrate. All other main cultures were inoculated directly from the preculture.

### Measurement of respiration activity

To monitor the respiration activity of the microorganisms noninvasively in shake flasks, an in-house built Respiration Activity Monitoring System (RAMOS) was used. This device can measure the oxygen transfer rate (OTR) and carbon dioxide transfer rate online and calculate the respective respiratory quotient (RQ) [28]. The OTR in MTPs was monitored using an in-house built Micro Transfer-Rate Online Measurement (µTOM) device [29]. A commercial version of the RAMOS device can be obtained from HiTec Zang GmbH (Herzogenrath, Germany) or Adolf Kühner AG (Birsfelden, Switzerland).

### Offline sample analytics

Samples from shake flask cultivations were drawn at the end of the cultivation from the respective RAMOS flasks. Samples from MTP cultivations were drawn from offline plates cultured in parallel to the MTP in the µTOM device in the same shaker. This approach was chosen, as direct sampling would have interfered with the µTOM measurement. Due to the small sample volume of the MTP experiments, three biological replicates were pooled before sample preparation. To still provide standard deviations for the product concentration, pooling of samples was performed thrice, i.e., nine wells were used for each measurement.

For measurements of the OD_600_, culture broth was diluted with 9 g/L NaCl in a range of 0.1–0.4 and measured with a Genesys 20 spectrophotometer (Thermo Fisher Scientific Inc., Waltham, Massachusetts, United States) using 1.6 mL Rotilabo cells (Carl Roth GMbH + Co. KG).

To quantify sucrose, glucose, fructose, and malic acid, the samples were analyzed via HPLC. HPLC samples from each shake flask were prepared via centrifugation of 1 mL of culture broth at 18000 *g* for 5 min and subsequent sterile filtration. HPLC samples from MTPs were centrifuged at 2680 *g* for 10 min and subsequently sterile filtered. A Thermo Fisher Ultimate 3000 (Thermo Fisher Scientific, Waltham, Massachusetts, United States) combined with a Rezex ROA-Organic Acid H + (8%) LC Column 300 × 7.8 mm (Phenomenex, Inc., Torrance (CA), United States) and a RefractoMax 520 RI detector (Thermo Fisher Scientific, Waltham, Massachusetts, United States) was used. As a mobile phase, sulphuric acid with a flow velocity of 0.8 mL/min at 20 °C was applied. For experiments investigating the buffer concentration and the influence of the carbon source, 25 mM sulphuric acid was used. For experiments investigating secondary substrate limitations, 50 mM sulphuric acid was used. The increase of malic acid concentration in MTPs during the cultivation due to evaporation was accounted for by developing a correlation between cultivation time and malic acid concentration c_regr_ (t) (Additional file 1: Figure S1). If not stated otherwise, the same experimental conditions as in the experiments investigating the secondary substrate limitations were used. The evaporation was investigated in an abiotic experiment and no sucrose was added to the medium. Instead, 10 g/L malic acid was supplied at the beginning of the experiment. HPLC samples were drawn during the cultivation and the malic acid concentration was measured. A corrected malic acid titer c_corr_ [g/L] was calculated based on the measured malic acid titer c_meas_ [g/L] of the limitation experiment. The evaporation factor between the beginning of the experiment t_0_ [h] and the sampling time point t_sample_ [h] is determined by the regression curve c_regr_ (t) [g/L]:

1 $${c}_{corr}={c}_{meas}\frac{{c}_{regr}({t}_{0})}{{c}_{regr}({t}_{sample})}$$

All final malic acid titers of MTP experiments were corrected with Eq. 1.

Samples for the analysis of osmolality were prepared as specified for HPLC samples and analyzed with a Gonotec Osmomat 3000 Single-Sample Freezing Point Osmometer (Gonotec Meß- und Regeltechnik GmbH, Berlin, Germany).

### Elemental composition of cell biomass

Cell biomass for elemental analysis was cultivated in shake flasks as described above using adapted Verduyn medium for unlimited growth. To achieve the respective limitation, the nitrogen or phosphate concentration were reduced to 1.5 g/L NH_4_Cl or 0.125 g/L KH_2_PO_4_, respectively. The cells were harvested by centrifugation with 1780 *g* at 4 °C for 10 min and washed twice with 9 g/L NaCl. The obtained cell pellets were dried at 60 °C for 48 h. The elemental composition of the cell biomass was analyzed by the Fraunhofer Institute for Applied Polymer Research IAP (Potsdam, Germany). C, H, N, S, and O analyses were performed in technical triplicates using a FlashEA 1112 Elemental Analyzer Series CHNS/O with MAS200R autosampler (Thermo Finnigan LLC, San Jose (CA), United States). The elements P and Mg were determined in technical duplicates using optical emission spectrometry with inductively coupled plasma (ICP OES) (Optima 2100 DV, Perkin-Elmer, Waltham, Massachusetts, United States). Approximately 10–50 mg sample were dissolved in 5 ml nitric acid in a microwave digestion system Start 1500 (MWS Mikrowellensysteme-Vertriebs GmbH, Leutkirch, Germany). The digestions were performed as duplicates. The clear solutions were diluted to 50 ml with ultrapure water and then injected into the ICP OES.

## Results and discussion

### Characterization of growth and malic acid production on molasses and pure sugars

For a successful production process based on sugar-rich residue streams, the ability of the organism to metabolize various carbon sources is crucial. Before investigating the suitability of beet molasses, the exemplary complex substrate of this study, suitable experimental conditions must first be established. Maintaining a stable pH value throughout the cultivation is essential for malic acid production with *U. trichophora*, as product formation is strongly hindered by low pH values [30]. Therefore, buffer systems are commonly applied in small-scale experiments without automatic pH control. MES buffer was previously reported to be suitable for carboxylic acid production with Ustilaginaceae, but was insufficient under the chosen conditions (0.1 M MES, initial pH value 6.5) [12, 26]. To overcome these limitations, the initial pH value was adjusted to 7.2, and buffer concentrations from 0.1 to 0.4 M were investigated, while maintaining a constant osmolality via the addition of respective amounts of NaCl (Additional file 1: Table S3, Figure S2). A buffer concentration of 0.3 M proved to be sufficient for efficient malic acid production and was therefore applied for all following experiments. The addition of NaCl was hereafter omitted.

**Fig. 1:**
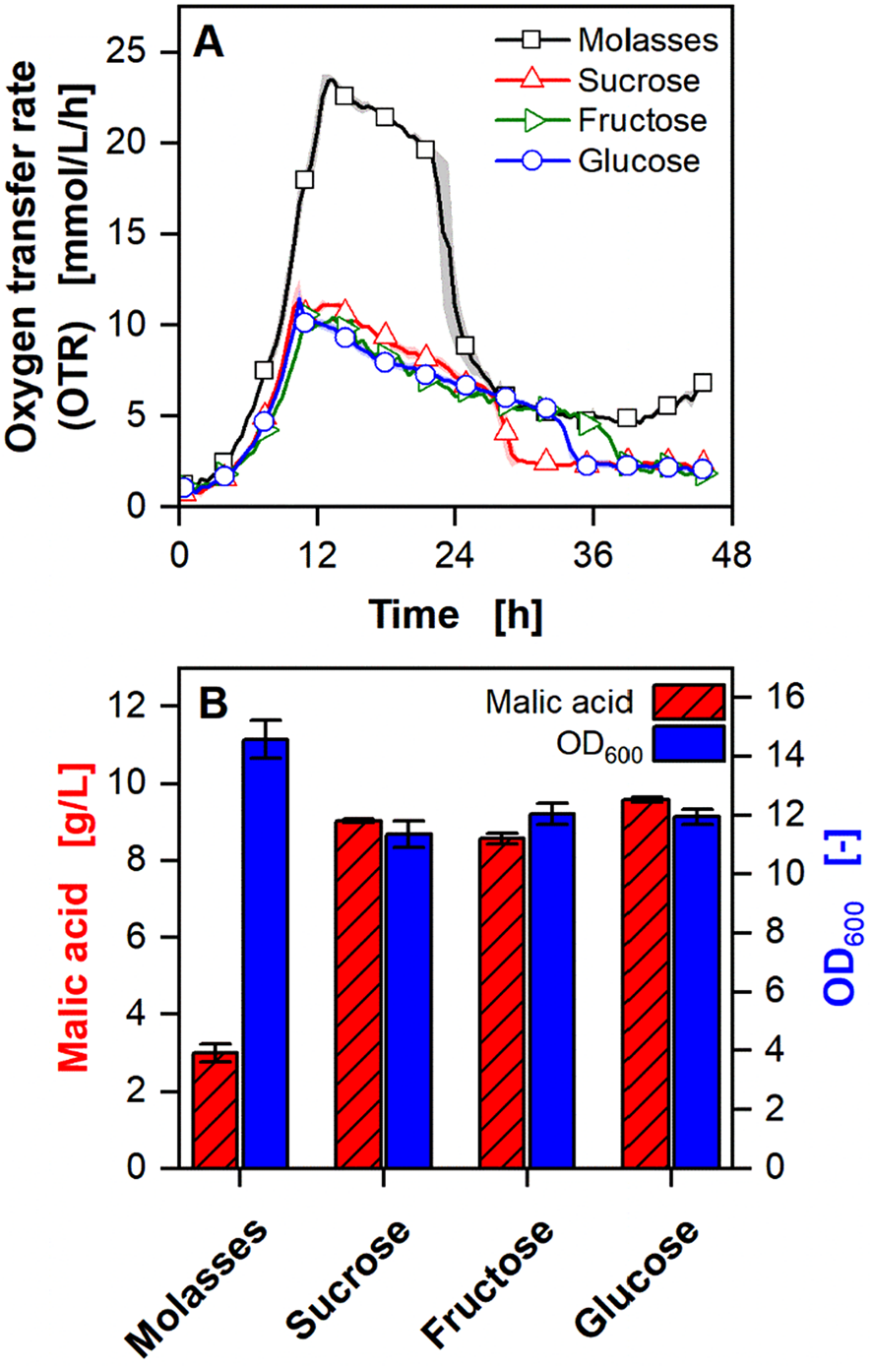
Malic acid production on molasses, sucrose, fructose, and glucose. **A** Online data of OTR. For clarity, only every fifth data point over time is represented as a symbol. Lines are drawn through all measuring points. Shadows indicate the minimum and maximum values of biological duplicates. For fructose, only a unicate is shown. **B** Malic acid concentration and OD_600_. Samples were drawn at the end of the cultivation. Error bars indicate the minimum and maximum values of biological duplicates. Cultivation conditions: *U. trichophora*, RAMOS device, 250 mL shake flasks, modified Verduyn medium (25 g/L sucrose, 26 g/L glucose, 26 g/L fructose, or molasses equivalent to 25 g/L sucrose), 0.8 g/L NH_4_Cl, 0.3 M MES (pH 7.2), T = 30 °C, n = 350 rpm, d_0_ = 50 mm, V_L_ = 20 mL, OD_600,start_ = 0.1 [–]

Beet molasses consists mainly of sucrose [17, 19]. To investigate growth and malic acid production on molasses with *U*. *trichophora*, C-mol equivalent amounts of sucrose, glucose, fructose, and molasses were supplied (Fig. 1). Molasses is commonly pretreated for fermentations [20, 31]. In this study, untreated molasses was used.

The OTR curves correspond to the typical trajectory for nitrogen-limited *Ustilago* sp. cultivations (Fig. 1A) [32–34]. In the beginning, the OTR increases exponentially due to the exponential growth of the organism. After nitrogen is depleted, a decreasing OTR plateau is formed, indicating the secondary substrate limitation. During this phase, the product malic acid is formed. The OTR then drops sharply after the complete consumption of the carbon source. No sugar could be detected at the end of the cultivations via HPLC. The OTR on all pure sugars is comparable during growth and production phase (Fig. 1A). Sucrose is depleted first, followed by glucose, and lastly fructose. For molasses, the onset of nitrogen limitation is delayed, compared to the pure sugars. After a slightly faster and longer growth phase, a shorter and higher OTR plateau during the production phase is visible. The maximum OTR reached on molasses is twice as high as on pure sucrose. The final OD_600_ and malic acid concentrations were comparable for all pure sugars (Fig. 1B). The final OD_600_ on molasses is 28% higher than on pure sucrose, which does not correspond to the observed difference in OTR. A possible explanation might be the production of additional biomass even after nitrogen is depleted, as already observed by Klement et al*.* [32]. Even though nitrogen limitation sets in earlier with pure sugars, continued growth would diminish the difference between the cultivations. Significantly less malic acid was detected for molasses. The deviating behavior on molasses can be attributed to the presence of complex medium components, such as proteins and amino acids, leading to faster growth and higher biomass concentrations [18, 19]. As more of the carbon source is used for biomass production, the production phase is shortened, resulting in significantly lower malic acid concentrations (Fig. 1B).

Prior studies on the metabolism of sucrose by *Ustilago* sp. revealed the action of an extracellular invertase, which hydrolyzes the disaccharide into the respective monomers glucose and fructose [18]. However, conflicting results have been published on the use of the three sugars for carboxylic acid production by *Ustilago* sp. Helm et al*.* reported reduced itaconic acid production on sucrose with *Ustilago maydis* and *Ustilago cynodontis* due to slow sucrose hydrolysis and reduced production rates on fructose [18]*.* Niehoff et al. also observed a lower itaconic acid titer on sucrose for *U. cynodontis*, but comparable production on glucose and fructose [33]. In contrast to the studies on itaconic acid, the malic acid concentrations were in a comparable range for all three pure sugars for *U. trichophora* (Fig. 1B). No limitation of sucrose or fructose utilization could be observed, which makes the residue stream molasses an attractive alternative feedstock for malic acid production. However, high intrinsic nitrogen concentrations pose an inherent challenge for the use of molasses for production processes triggered by nitrogen limitation. In the following, a suitable screening procedure will be developed to find alternative secondary substrate limitations for the production of malic acid.

### Investigation of potential secondary substrate limitations on defined minimal medium with pure sucrose

To clearly differentiate between the different secondary substrate limitations to be investigated, an adapted Verduyn medium for unlimited growth was developed as a control. For this, the concentrations of NH_4_Cl, KH_2_PO_4,_ and MgCl_2_ were sufficiently increased. No secondary substrate limitation is visible in the OTR and no malic acid is formed using the adapted Verduyn medium for unlimited growth (Additional file 1: Figure S3A) [34]. As the next step, an OTR-based scale-down was performed, to enable high-throughput screening in MTPs (Additional file 1: Figure S3B). For this purpose, the µTOM was used, enabling measurement of the OTR of each well in shaken 96-well MTPs [29]. The OTRs of MTP wells with a V_L_ of 0.1 mL and shake flasks showed a very similar progression, indicating a successful scale-down.

**Fig. 2:**
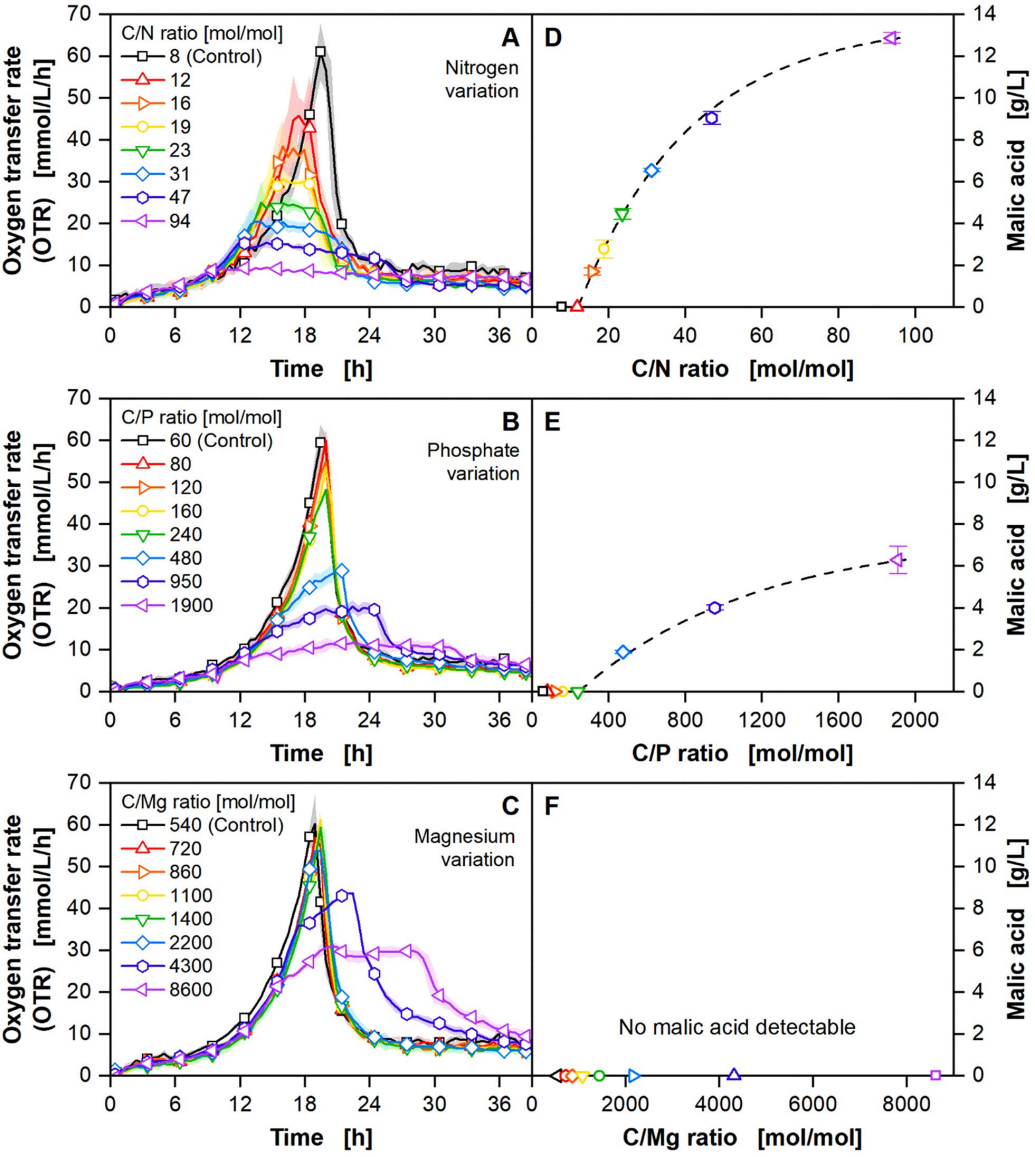
Impact of secondary substrate limitations on fermentations with pure sucrose. Influence of decreasing concentrations of nitrogen, phosphorus, and magnesium on **A**–**C** OTR and **D**–**F** malic acid production. **A**–**C** For clarity, only every fifth data point over time is represented as a symbol. Lines are drawn through all measuring points. Shadows indicate standard deviation of biological triplicates. **D**–**E** Samples were drawn after the sugar was fully consumed, as indicated by a drop in the OTR. Error bars indicate standard deviation of biological triplicates. Corresponding yields and space–time yields are provided in Additional file 1: Figure S5. Cultivation conditions: *U. trichophora*, µTOM device, 96 round deep well plates, modified Verduyn medium [25 g/L sucrose, 0.3 M MES (pH 7.2)], T = 30 °C, n = 1000 rpm, d_0_ = 3 mm, V_L_ = 100 µL, OD_600,start_ = 0.1 [–]. **A**, **D** 0.5–6 g/L NH_4_Cl, 0–6.6 g/L NaCl, 2 g/L KH_2_PO_4_, 0.4 g/L MgSO_4_ **B**, **E** 6 g/L NH_4_Cl, 0.06–2 g/L KH_2_PO_4_, 0–1.1 g/L KCl, 0.4 g/L MgSO_4_ **C**, **F** 6 g/L NH_4_Cl, 2 g/L KH_2_PO_4_, 0.025–0.4 g/L MgSO_4_, 0–0.23 g/L Na_2_SO_4_

Nitrogen, phosphate, magnesium, and sulphate limitation were screened for their influence on growth and malic acid production. For a first investigation of the secondary substrate limitations, defined minimal medium containing pure sucrose as carbon source was used. Based on the adapted Verduyn medium for unlimited growth, the concentration of each secondary substrate was gradually reduced. All secondary substrates were added as salts. When using MgSO_4_, replacing the respective counterion was necessary to investigate magnesium and sulphate limitation separately. Additionally, the respective counterions were added for KH_2_PO_4_ and NH_4_Cl, to avoid potential limitation of potassium and chloride. Potassium and chloride were not investigated in this study due to their abundance in molasses [18]. The µTOM device was used to monitor the influence of the secondary substrate limitations on the respiratory activity. Malic acid concentrations were determined at the end of the cultivation via HPLC. Results for nitrogen, phosphate, and magnesium limitation are displayed in Fig. 2. Sulphate limitation can be found in Additional file 1: Figure S4A. Due to the low sampling volume, the final biomass concentration could not be measured offline. As Klement et al. demonstrated a strict correlation between biomass formation and maximal reached OTR for *U. maydis* [32], biomass formation can, however, still be evaluated qualitatively from the respiratory activity measured online.

The state-of-the-art nitrogen limitation was used as a reference. Starting from the unlimited medium, the amount of NH_4_Cl was gradually decreased, resulting in a higher C/N ratio (Fig. 2A). Lower nitrogen concentrations resulted in an earlier onset of the nitrogen limitation and thus a longer production phase, as indicated by the height and length of the respective OTR plateau. The maximal OTR reached for the cultivation with the highest C/N ratio of 94 was only 15% of the unlimited control, implying that biomass formation was severely impacted by increasing nitrogen limitation. The highest NH_4_Cl concentration of 6 g/L negatively affected the growth rate, as indicated by the lower slope of the OTR at a C/N ratio of 8. Similar observations were already made for *U. maydis* and *U. vetiveriae* [32, 35]. Although secondary substrate limitation was already seen in the OTR for a C/N ratio of 12 mol/mol, malic acid was only detected from a C/N ratio of 16 mol/mol onwards (Fig. 2D). The malic acid concentration plotted against the C/N ratio shows the characteristic shape of a saturation curve. The highest malic acid concentration reached was 12.9 ± 0.3 g/L with a yield of 0.54 ± 0.02 g/g for a C/N ratio of 94. A complete overview of all yields and space–time yields is given in Additional file 1: Figure S5A and B. Carboxylic acid production provides an ecological advantage in the competition with other microorganisms through a drop in pH value and liberation of micronutrients through chelating [24, 36]. It is commonly observed as a stress response to nutrient starvation in fungi [37]. For some biosynthetic pathways, however, mere growth limitation is not sufficient to trigger organic acid production [38]. Organic acid synthesis has already been studied in detail for the model organism *U. maydis*. Itaconic acid, for example, is only produced under nitrogen limitation, due to specific nitrogen-sensitive promoters from the itaconate cluster [38]. Phosphate limitation does, however, lead to the accumulation of several unspecified organic acids [32].

To gain further insights into the effect of phosphate limitation on malic acid production with *U. trichophora,* an experiment with phosphate limitation was performed equally to nitrogen limitation. Starting from the unlimited medium, the amount of KH_2_PO_4_ was gradually decreased. From a carbon to phosphate (C/P) ratio of 240 onwards, phosphate limitation becomes visible in the OTR (2B). The shape of the OTR curves, however, clearly deviates from the nitrogen-limited cultures. Instead of an abrupt cessation of growth after the exponential growth phase, the growth rate slowly decreases for the phosphate-limited cultures. For the two lowest C/P ratios of 950 and 1900, a plateau of constant OTR is formed after approximately 20 h. Malic acid was detected from a C/P ratio of 480 onwards. Once more, the graph of malic acid concentration in relation to the C/P ratio displays the typical pattern of a saturation curve. The highest malic acid concentration reached was 6.3 ± 0.7 g/L with a yield of 0.25 ± 0.03 g/g for a C/P ratio of 1900. A complete overview of all yields and space–time yields is given in Additional file 1: Figure S5C and D. To date, no data on phosphate limitation with *U. trichophora* has been published. However, the production of malic acid, citric acid, and itaconic acid under phosphate limitation has already been demonstrated for *Aspergillus* sp. [39–41]. A strong effect of phosphate limitation on glycolysis, early TCA metabolites, such as citric acid, isocitric acid, and oxoglutaric acid, and oxygen consumption has also been reported for *Saccharomyces cerevisiae* [42]. The distinct trajectories of the OTR curves under phosphate limitation resemble those previously overserved for *Escherichia coli* and *Hansenula polymorpha* [43, 44]. The observed decrease in growth rate with decreasing phosphate concentrations could be attributed to several effects. For *S. cerevisiae*, the growth kinetic follows the Monod relationship, resulting in lower growth rates for lower extracellular phosphate concentrations [45]. Furthermore, internal phosphate pools may be used for further growth after depletion of extracellular phosphate, as observed for *H. polymorpha* or *Corynebacterium glutamicum* [44, 46]. As a result, the cells reduce their phosphorus content.

To investigate magnesium limitation, the amount of MgSO_4_ was gradually decreased, while supplementing Na_2_SO_4_ to the medium. Secondary substrate limitation is visible from a carbon to magnesium (C/Mg) ratio of 4300 onwards (Fig. 2C). The OTRs for the limited cultures first follow the exponential growth of the unlimited cultures. With the onset of the limitation, the slope of the OTR abruptly decreases. A plateau of constant OTR is formed for a C/Mg ratio of 8600. For a C/Mg ratio of 4300, the OTR decreases before a plateau is reached due to carbon depletion. No malic acid was detected for magnesium-limited conditions. Literature on the effect of magnesium limitation on organic acid production with fungi is scarce. Lipid accumulation, which is also commonly triggered by nutrient starvation, was observed for *Yarrowia lipolytica* under combined nitrogen and magnesium limitation [25]. Magnesium plays an important role as an enzyme cofactor for many glycolytic enzymes, in the stimulation of several important enzymes from the TCA cycle and the activation of ATP synthesis in the mitochondria [47, 48]. Free Mg^2+^-ions directly influence the activity of the rate-limiting enzyme of the TCA cycle [49, 50]. This may explain why the TCA intermediate malic acid is not detectable during magnesium-limited conditions.

To achieve sulphate limitation, the amount of MgSO_4_ was gradually decreased, while supplementing MgCl to the medium. For the calculation of the C/S ratio, the total amount of sulphate added by MgSO_4_, FeSO_4_, ZnSO_4_ and CuSO_4_ was used. No secondary substrate limitation was visible, even for the lowest carbon to sulphate (C/S) ratio of 6500 (Additional file 1: Figure S4A). No malic acid could be detected via HPLC. The results indicate a potential sulphate excess due to intracellular storage during normal growth conditions [51].

In literature, the nutritional requirements of microorganisms are often calculated based on their elemental composition [52]. This mathematical shortcut would be able to replace the presented screening, saving time and resources. The threshold for secondary substrate limitation would then be calculated based on parameters generally obtainable from literature or established analytics. A simple mass balance applying the microorganism’s biomass yield and elemental composition is used. For this, the elemental composition of *U. trichophora* during exponential growth was analyzed (Table 1A).

**Table 1:** Elemental composition of *U.* *trichophora* during exponential growth, under nitrogen limitation and under phosphate limitation

Growth conditions	Carbon [wt%]	Hydrogen [wt%]	Oxygen [wt%]	Nitrogen [wt%]	Phosphate [wt%]	Sulphate [wt%]	Magnesium [wt%]
Unlimited **A**	40.1 ± 0.7	6.51 ± 0.31	31.1 ± 0.8	7.95 ± 0.11	2.35 ± 0.13	0.343 ± 0.006	0.241 ± 0.018
Nitrogen limitation **B**	47.1 ± 1.1	7.53 ± 0.13	34.6 ± 2.9	3.96 ± 0.18	1.68 ± 0.10	0.177 ± 0.021	0.147 ± 0.010
Phosphate limitation **C**	49.1 ± 3.1	7.49 ± 0.38	33.6 ± 0.7	7.48 ± 0.60	0.51 ± 0.09	0.337 ± 0.021	0.064 ± 0.008

Uncertainties indicate standard deviation of technical triplicates for carbon, hydrogen, oxygen, nitrogen, and sulphate, and minimum and maximum of technical duplicates for phosphate and magnesium. Cultivation conditions: RAMOS device, 250 mL shake flasks, adapted Verduyn medium for unlimited growth [25 g/L sucrose, 0.4 g/L MgSO_4_, 0.3 M MES (pH 7.2)], T = 30 °C, n = 350 rpm, d_0_ = 50 mm, OD_600,start_ = 0.1 [-]. **A** V_L_ = 10 mL, 6 g/L NH_4_Cl, 2 g/L KH_2_PO_4_ **B** V_L_ = 20 mL, 1.5 g/L NH_4_Cl, 2 g/L KH_2_PO_4_ **C** V_L_ = 20 mL, 6 g/L NH_4_Cl, 0.125 g/L KH_2_PO_4_

The thresholds for secondary substrate limitation of nitrogen and phosphate (C/E_thres_) [mol/mol] were subsequently calculated according to Eq. 2 using the biomass yield of the organism on sucrose (Y_XSuc_) [g/g], the percentage of the respective secondary substrate in the biomass during exponential growth (y_E,X_) [wt%], and the molecular weight of sucrose (MW_Suc_) [g/mol] and the respective secondary substrate (MW_E_) [g/mol]:

2 $$C/{E}_{thres}= \frac{M{W}_{E}}{{Y}_{XSuc}*\frac{{y}_{E,X}}{100}*M{W}_{Suc}}$$

Y_XSuc_ was estimated based on a previously published biomass yield of 0.47 g/g for *U. maydis* on glucose [53]. Medium compositions with a C/N or C/P ratio above the calculated thresholds of 13 and 98, respectively, were expected to result in secondary substrate limitation. Consequently, only the media with a C/N ratio of 8 and 12 or a C/P ratio of 60 and 80 should have led to unlimited growth (Fig. 2A and B). For nitrogen limitation, only a slight reduction of the maximal reached OTR was visible for a C/N ratio of 12 compared to the unlimited control with a C/N ratio of 8 (Fig. 2A). Product formation was first detected for a C/N ratio of 16. Therefore, the calculated prediction agrees well with the experimental data. For phosphate limitation though, a clear impact on the OTR was visible only for much higher C/P ratios. Malic acid was first detected at a C/P ratio of 480 (Fig. 2E), which represents only 20 mol% of the limitation threshold. In addition, according to calculation, *U. trichophora* would be magnesium limited for C/Mg ratios of 700 onwards, although a noticeable impact on the OTR was only visible from a C/Mg ratio of 4300 onwards (Fig. 2C). For sulphate, even C/S ratios of 6500 were not sufficient to show an impact on the OTR (Additional file 1: Figure S4A), although the calculated threshold was at a C/S ratio of 750. Consequently, calculated limitation thresholds only have limited predictive power for *U. trichophora.*

To gain further insights into the metabolic impact of secondary substrate limitations, the elemental composition of *U. trichophora* under nitrogen and phosphate limitation was analyzed (Table 1B and C). The elemental compositions reveal changes in the cell’s elemental content, emphasizing the organism's ability to adapt to changing environments. During nitrogen limitation, the nitrogen content decreased by 50%, compared to exponential growth. A significant decrease was also observed for phosphate, sulphate, and magnesium. The carbon and hydrogen content increased significantly. During phosphate limitation, the phosphate content decreased by 78%. However, no effect on nitrogen or sulphate content was detected. Only for magnesium, a notable decrease was measured. As for nitrogen limitation, the carbon and hydrogen content increased significantly for phosphate limitation. The limitation threshold was again calculated based on the corrected real elemental composition under phosphate limitation, while neglecting a potential impact on biomass yield. With this correction, the predictions and the observed data are more in agreement. Phosphate limitation should then only be visible for a C/P ratio greater than 450, as provided by the last three limitation steps (C/P 480, 950, 1900) (Fig. 2B). Indeed, product formation was first detected at a C/P ratio of 480.

The previous results indicate a very different mode of action for nitrogen and phosphate limitation. Fungal cells react to nitrogen limitation by degradation of nitrogen-containing compounds and reduction of protein synthesis [37], consequently leading to reduced nitrogen contents of the microbial biomass [32, 54]. For *U. maydis*, a dilution of intracellular nitrogen was observed, due to an additional reproduction cycle after extracellular nitrogen was depleted [32]. The carbon and hydrogen content increased due to the formation of intracellular hydrocarbons. Reproduction rates were much slower than during unlimited growth and no increase in metabolic activity was visible as indicated by the OTR. On the other hand, *Corynebacterium glutamicum* was able to maintain its growth rate, when extracellular phosphate was depleted, by reducing the internal phosphate content [46]. Many species of fungi can store excessive phosphate as polyphosphate in their vacuoles [42, 55, 56]. Under phosphate-limited conditions, the stored polyphosphate is degraded and thus buffers the cytosolic phosphate levels. Indeed, wild-type *U. maydis* can store polyphosphate under conditions of high extracellular phosphate [57]. Polyphosphate is also known as an efficient chelator of Mg^2+^ ions, leading to an accumulation of magnesium inside the vacuoles and a direct correlation between intracellular magnesium and phosphate levels [58, 59]. Taking this study’s results regarding elemental composition and OTR into account, it is hypothesized that *U. trichophora* can buffer a depletion of extracellular phosphate using its internal phosphate storage to resume growth.

The preceding findings lead to an important conclusion. No reliable statement on the onset of secondary substrate limitations can be made via calculation with literature values of elemental biomass composition alone. Microorganisms have evolved to cope with fluctuating nutrient availability, which enables them to buffer nutrient scarcity through internal storage molecules. Furthermore, malic acid production must be evaluated separately from secondary substrate limitation, as successful secondary substrate limitation does not guarantee product formation. The presented screening procedure thus offers an important method to reliably identify suitable secondary substrate limitations.

### Investigation of the suitability of secondary substrate limitations on molasses-based complex medium

**Fig. 3:**
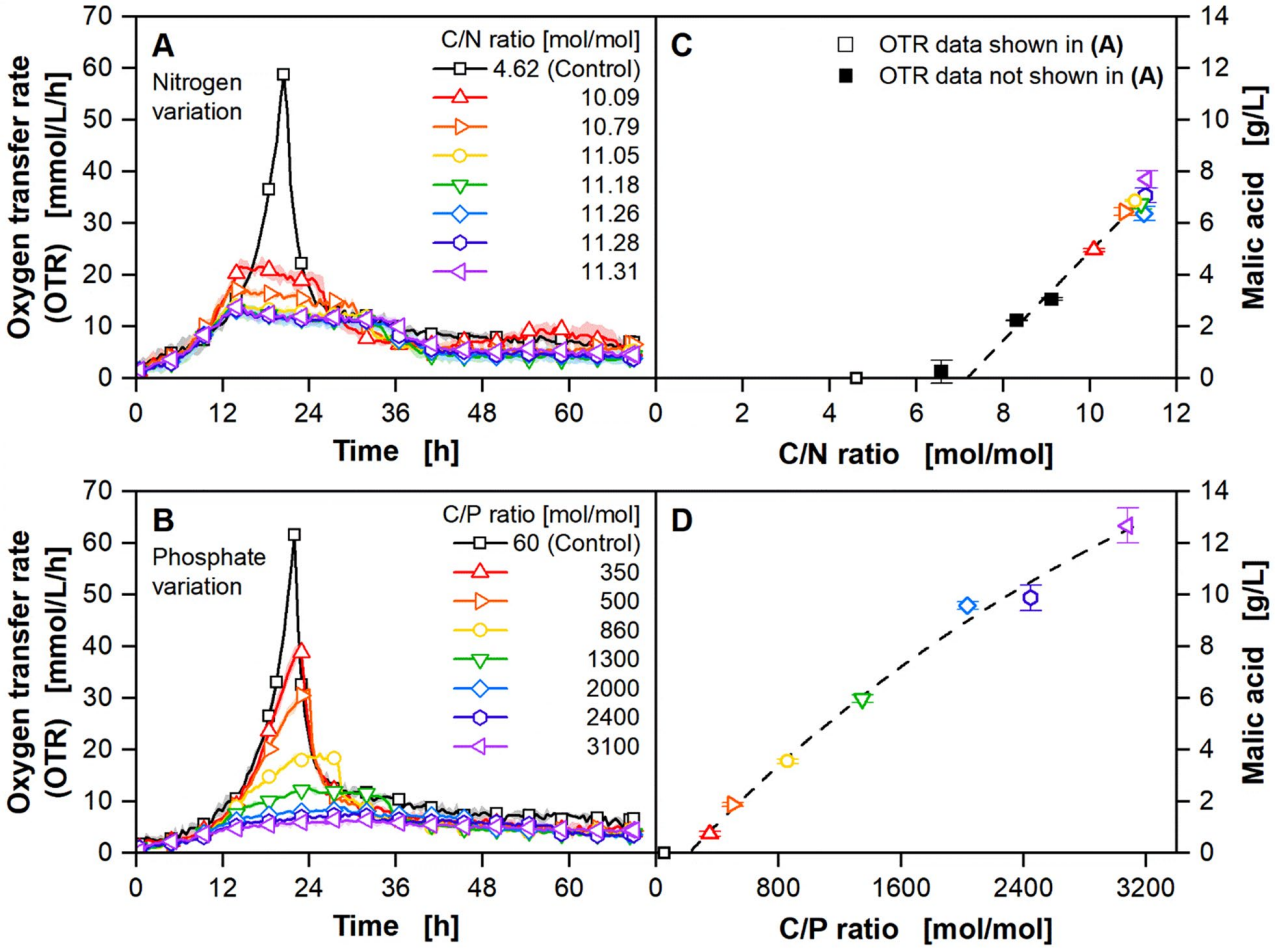
Impact of secondary substrate limitations on fermentations with the complex substrate molasses. Influence of decreasing additional nitrogen, phosphorus, and magnesium concentrations on **A**, **B** OTR and **C**, **D** malic acid production. **A**, **B** For clarity, only every ninth data point over time is represented as a symbol. Lines are drawn through all measuring points. Shadows indicate standard deviation of biological triplicates. **C**, **D** Samples were drawn after the sugar was fully consumed, as indicated by a drop in the OTR. Error bars indicate standard deviation of biological triplicates. **C** Open symbols indicate samples, which were taken from the experiment shown in **A**. Filled symbols indicate samples, which were taken from an additional experiment investigating different C/N ratios. Corresponding yields and space–time yields are provided in Additional file 1: Figure S7. The respective C/E ratios were calculated based on the total amount of secondary substrate in molasses [18], and the respective additional salts. Cultivation conditions: *U. trichophora*, µTOM device, 96 round deep well plates, modified Verduyn medium [molasses equivalent to 25 g/L sucrose, 0.3 M MES (pH 7.2)], T = 30 °C, n = 1000 rpm, d_0_ = 3 mm, *V*_L_ = 100 µL, OD_600,start_ = 0.1 [–]. **A**, **C** 0–6 g/L NH_4_Cl, 0–6.6 g/L NaCl, 2 g/L KH_2_PO_4_, 0.4 g/L MgSO_4_ **B**, **D** 6 g/L NH_4_Cl, 0–2 g/L KH_2_PO_4_, 0–1.1 g/L KCl, 0.4 g/L MgSO_4_

In the prior section, phosphate limitation was found to be suitable for malic acid production with *U. trichophora* on minimal medium with pure sucrose. This finding expands the previously known nitrogen limitation as a tool for efficient malic acid production. The applicability of the investigated limitations on the complex substrate beet molasses is investigated next. Instead of pure sucrose, untreated molasses was used as the carbon source (Fig. 3). Even though magnesium and sulphate limitation were not suitable for malic acid production with *U. trichophora*, they might still be of interest for other products or microorganisms. To evaluate whether the molasses’ composition allows for their limitation, both secondary substrates were also investigated in the following. Similar to the previous section, either the amount of additional nitrogen, phosphate, sulphate, or magnesium was gradually reduced based on the adapted Verduyn medium for unlimited growth. The according counterions were supplemented. No additional NH_4_Cl, KH_2_PO_4,_ or MgSO_4_ was added for the lowest carbon to secondary substrate (C/E) ratio. Both, secondary substrate derived from the molasses itself and from additional salts from the Verduyn medium, were considered in the calculation of the C/E ratio ratios. Further information on the calculation of the C/E ratios for the experiments on molasses is provided in the section “Materials and methods”. An exemplary overview of the concentrations and origins of nitrogen and phosphorus in the molasses-based medium can be found in Additional file 1: Figure S6. The investigation of their bioavailability will be covered in the next section. More detailed information on the carbon and nitrogen sources and the trace elements contained in molasses is provided by Helm et al. [18].

Nitrogen limitation was again used as a reference (Fig. 3A). Starting from the unlimited control, the amount of NH_4_Cl was gradually decreased, resulting in increasing C/N ratios. The typical trajectory for nitrogen-limited cultures can again be recognized for all cultivation conditions, except for the control. Significantly lower OTR plateaus were observed on molasses than on defined minimal medium with sucrose and with comparable C/N ratios. Even at the highest C/N ratio without any additional NH_4_Cl, the organism still grew to a maximal OTR of 14.3 mmol/L/h, due to the nitrogen supplied by the molasses. This condition reached the highest malic acid concentration of 7.7 ± 0.3 g/L with a yield of 0.34 ± 0.01 g/g. Figure 3C suggests a linear correlation between the malic acid concentration and the C/N ratio, in contrast to the observations made on defined minimal medium (Fig. 2D). A complete overview of all yields and space–time yields is given in Additional file 1: Figure S7A and B. A further comparison of the results from the limitation experiments on defined minimal medium and on complex medium will be given in the next section.

Phosphate limitation was performed as described previously. Starting from the unlimited control, the amount of KH_2_PO_4_ was gradually decreased (Fig. 3B). For all culture conditions except for the control with the lowest C/P ratio, the OTR curves show the trajectory of phosphate-limited cultures with a steady decrease of the growth rates. From a C/P ratio of 860 onwards, a plateau is formed at the end. Even at the lowest C/P ratio without any additional KH_2_PO_4_, the organism still grew to a maximal OTR of 6.4 mmol/L/h, due to the phosphate supplied by the molasses. This condition reached the highest malic acid concentration of 12.7 ± 0.7 g/L with a yield of 0.55 ± 0.03 g/g. Similar to the cultivations with nitrogen limitation, an approximately linear correlation between the malic acid concentration and the C/P ratio is observed (Fig. 3D). A complete overview of all yields and space–time yields is given in Additional file 1: Figure S7C and D.

For magnesium limitation, the amount of MgSO_4_ was gradually decreased (Additional file 1: Figure S8). Magnesium limitation was visible from a C/Mg ratio of 5100 onwards. In accordance with the data from the defined minimal medium, no malic acid was produced under magnesium limitation. However, the data suggest that molasses could possibly be used for other production processes triggered by magnesium limitation.

To achieve sulphate limitation, the amount of MgSO_4_ was gradually decreased (Additional file 1: Figure S4B). Due to high intrinsic sulphate concentrations of molasses, only a maximal C/S ratio of 106 could be reached. This corresponds to fivefold higher sulphate concentrations than used in the unlimited medium on sucrose (Additional file 1: Figure S4A), effectively preventing sulphate limitation.

The elemental composition of molasses is significantly influenced by the origin of sugar beets and the manufacturing process. The magnesium content is strongly influenced by the respective soil and fertilizer applied. Sugar beets grown on lime-rich soil may produce almost magnesium-free molasses [60]. In contrast, elements such as phosphate or sulphate are particularly affected by sugar manufacturing. Sugar is first extracted from sliced sugar beets with hot water. In the next step, the beet juice is clarified using Ca(OH)_2_ and CO_2_. Commonly, a sulphitation step follows afterwards [60, 61]. Phosphate is precipitated as insoluble calcium phosphate during clarification, causing the low phosphate content of molasses [61, 62]. Therefore, phosphate is often supplemented in microbial fermentations on molasses [15, 63]. Opposed to this, a large portion of nitrogen passes manufacturing unaltered in the form of proteins, amino acids, and betaine [17, 18, 60]. When no NH_4_Cl or KH_2_PO_4_ was supplemented to molasses-based medium, a 2.2-fold higher maximal OTR was observed for nitrogen limitation compared to phosphate limitation, indicating a higher biomass formation. This disparity can be attributed to the 270 mol/mol higher nitrogen content relative to phosphate in molasses, which inherently favors biomass accumulation under nitrogen-limiting conditions. Conversely, phosphate limitation yields lower biomass concentrations, which results in a 1.6-fold increased malic acid concentration with a yield of 0.55 ± 0.03 g malic acid per g sucrose. Malic acid can be produced via three pathways: the oxidative TCA cycle, the reductive TCA cycle, and the glyoxylate pathway [3]. The exact mechanism of malic acid production in *U. trichophora* is yet to be investigated [12]. When using the oxidative TCA cycle, the experimentally achieved yield corresponds to 70% of the theoretical maximal yield of 0.78 g malic acid per g sucrose (2 mol/mol). When using the reductive TCA cycle or the glyoxylate pathway, the percentage decreases to 35% or 53%, respectively. In general, obtained yields are lower than the theoretical maximum due to biomass growth, by-product formation, and maintenance [12]. Biomass formation under phosphate-limited conditions is already low, according to the OTR. Byproduct formation may play an important role, as a wild-type strain was used in this study. Strain engineering focusing on higher selectivity by disrupting by-product formation pathways has therefore the greatest potential to increase malic acid yields.

The impact of fermentation optimization on process economics was approximated using a current molasses price of 300 €/t [64]. With the maximum yields achieved under nitrogen and phosphate limitation, substrate costs of 1.58 €/kg and 0.97 €/kg malic acid were calculated, respectively. Based on previous techno-economic analyses for itaconic acid production with *U. maydis* on thick juice, substrate costs account for approximately 50% of the total costs [13]. Consequently, total costs of 3.2 €/kg malic acid were estimated when using the state-of-the-art nitrogen limitation. When using the novel phosphate limitation, total costs decrease to 2.0 €/kg. In 2023, the market price of malic acid was recorded at 2.8 €/kg [65].

### Comparison of minimal and complex medium and investigation of bioavailability of secondary substrates in molasses

**Fig. 4:**
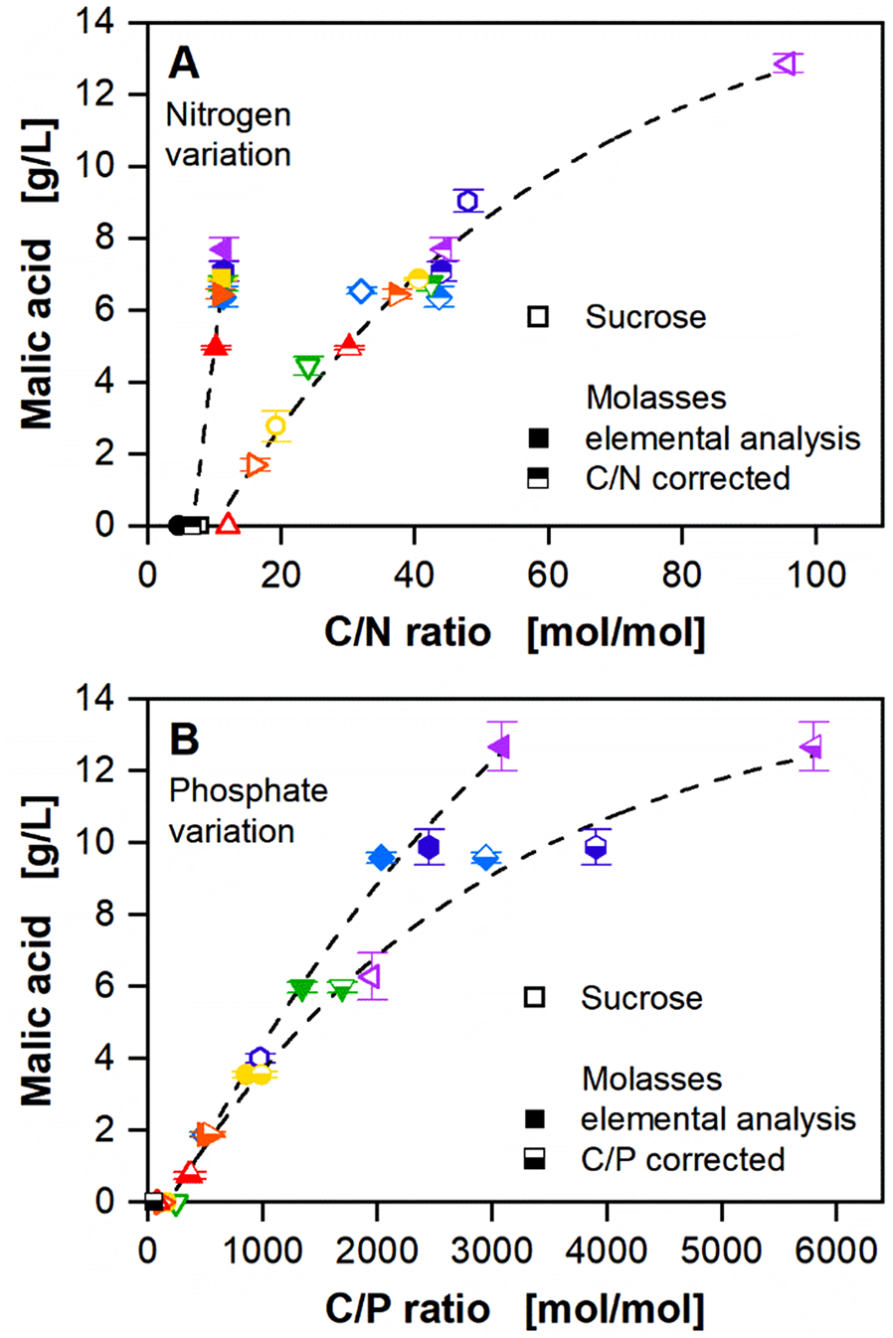
Comparison of malic acid production under nitrogen and phosphate limitation on sucrose and molasses*.* Complete data are shown in Fig. 2 and Fig. 3, the cultivation conditions are given in the respective figures. Error bars indicate standard deviation of biological triplicates. Open symbols indicate that sucrose was used as substrate. Filled symbols indicate that molasses was used as substrate and the respective C/N and C/P ratios were calculated based on the total amount of nitrogen and phosphorus in molasses, respectively (Helm et al*.,* 2023), and the respective additional salts. **A** Half-filled symbols indicate a corrected C/N ratio based on the amount of bioavailable nitrogen, as determined in Additional file 1: Figure S9, and the respective additional salts **B** Half-filled symbols indicate a corrected C/P ratio based on the amount of bioavailable phosphate, as determined in Additional file 1: Figure S10, and the respective additional salts

When comparing the OTR curves and the malic acid titers from the experiments on pure sucrose and molasses, deviations become immediately apparent (Fig. 2, Fig. 3). Firstly, OTR plateaus of comparable height were reached for significantly lower C/N ratios on molasses compared to sucrose (Fig. 2A, Fig. 3A). Furthermore, a clear difference of the curve trajectories, when plotting the malic acid concentration over the respective C/E ratio, was visible (Fig. 2D and E, Fig. 3C and D). Product formation on pure sucrose and molasses was therefore compared for the investigated C/N and C/P ratios (Fig. 4).

Previous studies already hypothesized that only part of the secondary substrates in complex substrates is bioavailable to microorganisms [16, 18]. According to the method developed by Niehoff et al*.* [33], the concentration of bioavailable nitrogen and phosphate were estimated. Based on the data on the defined minimal medium, a linear correlation of the maximal OTR of secondary substrate limited cultivations to the secondary substrate concentration supplied to the medium was determined (Additional file 1: Figure S9A and C, Figure S10A and C). For phosphate, only C/P ratios displaying a fully developed plateau were considered for evaluation (Additional file 1: Figure S10A). The correlation was subsequently used to calculate the concentration of bioavailable secondary substrate in molasses (Additional file 1: Figure S9B and D, Figure S10B and D). According to this method, only approximately 25% and 50% of all nitrogen and phosphate in molasses, respectively, can be assimilated by *U. trichophora*. The bioavailability of nitrogen in complex substrates is well investigated [52, 60]. Previous studies state that 15–55% of all nitrogen in molasses is bioavailable for *S. cerevisiae* [16]. Substrates with limited assimilation potential, such as betaine, have previously been documented as suboptimal nitrogen sources for *U. maydis* [66]. Data on the bioavailability of phosphate in molasses are scarce. The formation of insoluble phosphate precipitates such as ferric phosphate or struvite might hinder the complete assimilation of elemental phosphorus by *U. trichophora* [52, 67]. So far, the C/E ratios of the screening experiment on molasses were calculated based on the elemental analysis of molasses [18]. With the new estimations on bioavailability, the C/E ratios were recalculated. The final malic acid concentration was plotted over the corrected C/E ratio for both nitrogen and phosphate limitation (Fig. 4). Malic acid formation on sucrose and molasses now aligns on a single trend line, signifying the comparability of both substrates. Ideally, once suitable C/E ratios have been identified using defined minimal medium, simple calculations should be sufficient to evaluate the performance of any complex substrate. However, the simple mathematical shortcut based on the elemental composition of molasses reaches its limits. The limited bioavailability of the secondary substrates in molasses necessitates experimental screening.

## Conclusion and outlook

Reducing the substrate costs of a fermentation process is a crucial step in replacing petrochemical products with sustainable, biotechnological platform chemicals. Complex residue streams, such as nitrogen-rich molasses, pose challenges for processes requiring nitrogen limitation, which leads to suboptimal yields in Ustilaginaceae. Identifying suitable alternative feedstocks remains difficult, given the unpredictability of biochemical effects, the influence of internal storage mechanisms, and the incomplete bioavailability of secondary substrates in complex residues. This work bridges the gap between laboratory conditions and real-world residue streams by a promising microliter-scale screening method with online monitoring of microbial respiration. Initial investigations on a defined medium with pure sucrose assessed potential secondary substrate limitations for malic acid production with *U. trichophora*. Subsequently, effective limitations were applied to a medium containing untreated molasses. Phosphate limitation successfully led to malic acid production. Due to the low phosphate content of molasses, phosphate limitation was proven to be better suited than nitrogen limitation, achieving 1.6-fold higher malic acid concentrations by omitting all additional phosphate. Further analysis revealed an influence of internal polyphosphate storage and incomplete phosphate assimilation on phosphate limitation in *U. trichophora*, with only 50% of phosphate in molasses being bioavailable.

The intrinsic C/P ratio of molasses limits the maximum product yield, making product titer optimization critical for profitability. Due to the finite buffer capacity in shake-flask cultivations, the transition to fermenter scale is crucial for efficient pH control. Furthermore, metabolic engineering of the wild-type strain has great potential to further increase selectivity and productivity. The replacement of the state-of-the-art nitrogen limitation with the better suited phosphate limitation now paved the way for efficient malic acid production from molasses. This presents new opportunities for the utilization of molasses as a sustainable and cost-effective substrate for biobased platform chemical production.

### Supplementary Information

Additional file 1. **Table S1**: Concentration of salts used for experiments investigating secondary substrate limitations on sucrose-based minimal medium. **Table S2**: Concentration of salts used for experiments investigating secondary substrate limitations on molasses-based complex medium. **Figure S1**: Exponential regression of the malic acid increase in MTPs caused by evaporation. **Table S3**: Osmolality of medium used for the investigation of different buffer concentrations without adjustment with NaCl. **Figure S2**: Influence of elevated buffer concentrations on malic acid production. **Figure S3**: OTR-based scale-down of cultivation conditions from (A) shake flask to (B) MTP scale. **Figure S4**: No secondary substrate limitation was observed for the investigated sulphate concentrations. **Figure S5**: Effect of nitrogen and phosphate limitation on malic acid yield and space-time yield on sucrose. **Figure S6**: Concentration, origin and bioavailability of nitrogen and phosphorus in adapted Verduyn medium with molasses. **Figure S7**: Effect of nitrogen and phosphate limitation on malic acid yield and space-time yield on molasses. **Figure S8**: Impact of magnesium limitation on fermentations with the complex substrate molasses. **Figure S9**: Estimation of biologically available nitrogen in molasses. **Figure S10**: Estimation of biologically available phosphorus in molasses.

## Data Availability

The datasets supporting the conclusions of this article are included within the article and its additional file (Additional file 1.pdf: Table S1, Table S2, Figure S1, Table S3, Figure S2, Figure S3, Figure S4, Figure S5, Figure S6, Figure S7, Figure S8, Figure S9, Figure S10).

## Abbreviations

V_L_: Filling volume
T: Cultivation temperature
n: Shaking frequency
d_0_: Shaking diameter
MTP: Microtiter plate
MES: 2-(N-morpholino) ethanesulfonic acid
C/E ratio: Carbon-to-secondary substrate ratio
C/E_thres_: Threshold for secondary substrate limitation
OD_600_: Optical density
HPLC: High performance liquid chromatography
OTR: Oxygen transfer rate
RQ: Respiratory quotient
RAMOS: Respiration Activity Monitoring System
µTOM: Micro Transfer-Rate Online Measurement
TCA: Tricarboxylic acid
C/N ratio: Carbon-to-nitrogen ratio
C/P ratio: Carbon-to-phosphate ratio
C/Mg ratio: Carbon-to-magnesium ratio
C/S ratio: Carbon-to-sulphate ratio
Y_XSuc_: Biomass yield on sucrose
y_E,X_: Percentage of secondary substrate in biomass
MW: Molecular weight
