# Supplementary material for: Phosphate limitation enhances malic acid production on nitrogen-rich molasses with Ustilago trichophora
Source: Biotechnol Biofuels Bioprod. 2024 Jul 3;17:92. doi: 10.1186/s13068-024-02543-z (PMC11223335; doi:10.1186/s13068-024-02543-z)
Supplement: Supplementary file 1 — Additional file 1. Table S1: Concentration of salts used for experiments investigating secondary substrate limitations on sucrose-based minimal medium. Table S2: Concentration of salts used for experiments investigating secondary substrate limitations on molasses-based complex medium. Figure S1: Exponential regression of the malic acid increase in MTPs caused by evaporation. Table S3: Osmolality of medium used for the investigation of different buffer concentrations without adjustment with NaCl. Figure S2: Influence of elevated buffer concentrations on malic acid production. Figure S3: OTR-based scale-down of cultivation conditions from (A) shake flask to (B) MTP scale. Figure S4: No secondary substrate limitation was observed for the investigated sulphate concentrations. Figure S5: Effect of nitrogen and phosphate limitation on malic acid yield and space-time yield on sucrose. Figure S6: Concentration, origin and bioavailability of nitrogen and phosphorus in adapted Verduyn medium with molasses. Figure S7: Effect of nitrogen and phosphate limitation on malic acid yield and space-time yield on molasses. Figure S8: Impact of magnesium limitation on fermentations with the complex substrate molasses. Figure S9: Estimation of biologically available nitrogen in molasses. Figure S10: Estimation of biologically available phosphorus in molasses. [file 13068_2024_2543_MOESM1_ESM.pdf]

Additional file 1

Phosphate limitation enhances malic acid production on nitrogen-rich molasses with *Ustilago trichophora*

Luca Antonia Grebe[1], Philipp Georg Lichtenberg[1], Katharina Hürter[1], Eva Forsten[1], Katharina Miebach[1], Jochen Büchs[1], Jørgen Barsett Magnus[1]*

[1]AVT - Biochemical Engineering, RWTH Aachen University, Forckenbeckstraße 51, 52074 Aachen, Germany

* Correspondence: Prof. Dr.-Ing. Jørgen Magnus (jorgen.magnus@avt.rwth-aachen.de)

Table S1: Concentration of salts used for experiments investigating secondary substrate limitations on sucrose-based minimal medium.

Nitrogen			Phosphate		
C/N [mol/mol]	NH_4Cl [g/L]	NaCl [g/L]	C/P [mol/mol]	KH_2PO_4 [g/L]	KCl [g/L]
8	6.00	0.00	60	2.000	0.000
12	4.00	2.19	80	1.500	0.276
16	3.00	3.28	120	1.000	0.551
19	2.50	3.82	160	0.750	0.689
23	2.00	4.37	240	0.500	0.827
31	1.50	4.92	480	0.250	0.964
47	1.00	5.46	950	0.125	1.033
94	0.50	6.01	1900	0.063	1.068
**Magnesium**			**Sulphate**		
C/Mg [mol/mol]	$MgSO_4$ [g/L]	Na_2SO_4 [g/L]	C/S [mol/mol]	$MgSO_4$ [g/L]	$MgCl_2$ [g/L]
540	0.400	0.000	540	0.400	0.000
720	0.300	0.083	710	0.300	0.058
860	0.250	0.124	850	0.250	0.087
1100	0.200	0.165	1100	0.200	0.115
1400	0.150	0.207	1400	0.150	0.144
2200	0.100	0.248	2000	0.100	0.173
4300	0.050	0.289	3700	0.050	0.202
8600	0.025	0.310	6500	0.025	0.216

Table S2: Concentration of salts used for experiments investigating secondary substrate limitations on molasses-based complex medium.

Nitrogen			Phosphate		
C/N [mol/mol]	NH_4Cl [g/L]	NaCl [g/L]	C/P [mol/mol]	KH_2PO4 [g/L]	KCl [g/L]
4.62	6.00	0.00	60	2.00	0.000
10.09	0.50	6.01	350	0.30	0.937
10.79	0.20	6.34	500	0.20	0.992
11.05	0.10	6.45	860	0.10	1.047
11.18	0.05	6.50	1300	0.05	1.075
11.26	0.02	6.53	2000	0.02	1.091
11.28	0.01	6.54	2400	0.01	1.097
11.31	0.00	6.56	3100	0.00	1.102

Magnesium			Sulphate		
C/Mg [mol/mol]	$MgSO_4$ [g/L]	Na_2SO_4 [g/L]	C/S [mol/mol]	$MgSO_4$ [g/L]	$MgCl_2$ [g/L]
520	0.4	0	88.4	0.4	0
3000	0.05	0.20206098	103.2	0.05	0.28929065
5100	0.02	0.21938049	104.7	0.02	0.31408699
6600	0.01	0.22515366	105.2	0.01	0.32235244
7800	0.005	0.22804024	105.5	0.005	0.32648516
8800	0.002	0.2297722	105.6	0.002	0.3289648
9100	0.001	0.23034951	105.7	0.001	0.32979134
9500	0	0.23092683	105.7	0	0.33061789

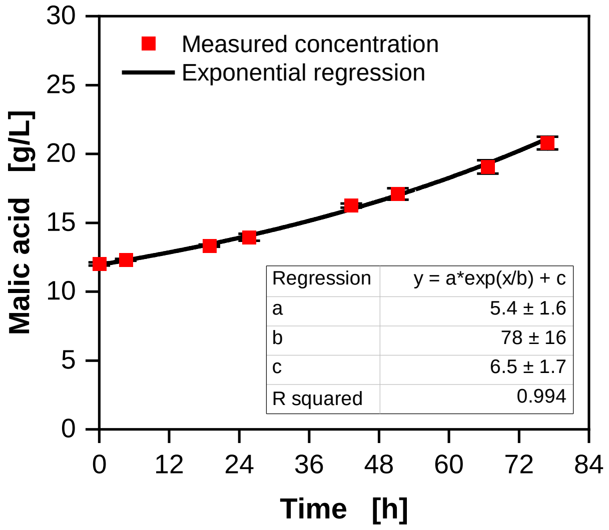

Figure S1: Exponential regression of the malic acid increase in MTPs caused by evaporation. Experimental conditions: 96 round deep well plates, modified Verduyn medium (10 g/L malic acid, 6 g/L NH_4Cl, 2 g/L KH_2PO_4, 0.4 g/L $MgSO_4$, 0.3 M MES (pH 7.2)), T = 30 °C, n = 1000 rpm, d_0 = 3 mm, V_L = 100 µL. Error bars indicate the standard deviation of triplicates.

Table S3: Osmolality of medium used for the investigation of different buffer concentrations without adjustment with NaCl.

MES buffer concentration [M]	Medium osmolality before adjustment [mOsmol/kg]
0.1	376
0.2	585
0.3	787
0.4	992

Modified Verduyn medium: 25 g/L sucrose, 0.8 g/L NH_4Cl, 0.1-0.4 M MES (pH 7.2).

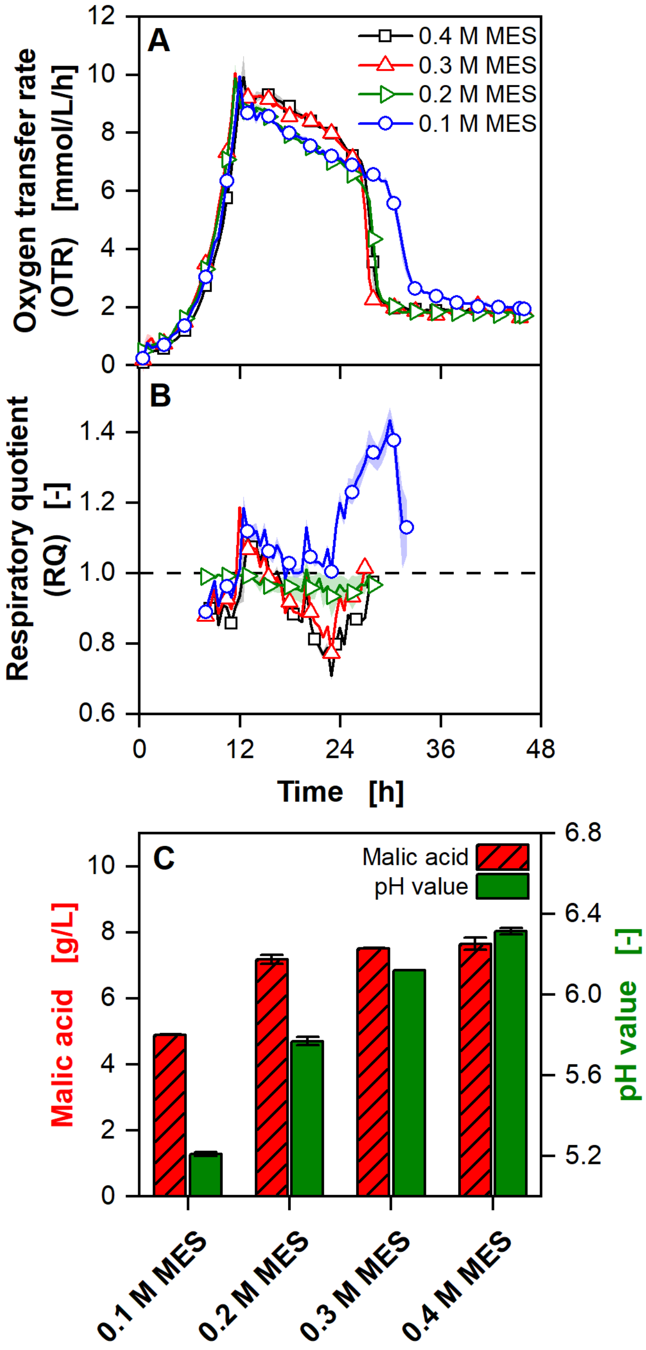

Figure S2: Influence of elevated buffer concentrations on malic acid production. (**A**) Online data of OTR. (**B**) Online data of RQ. Due to measurement inaccuracies at low OTR values, only RQ values for OTR values greater than 3 mmol/L/h are shown. (**A**), (**B**) For clarity, only every fifth data point over time is represented as a symbol. Lines are drawn through all measuring points. Shadows indicate the minimum and maximum values of biological duplicates. (**C**) Measured malic acid concentration and pH value. Samples were drawn at the end of the cultivation. Error bars indicate the minimum and maximum values of biological duplicates. Cultivation conditions: *U. trichophora*, RAMOS device, 250 mL shake flasks, modified Verduyn medium (25 g/L sucrose, 0.8 g/L NH_4Cl, 0.1-0.4 M MES (pH 7.2), 0-18 g/L NaCl), T = 30 °C, n = 350 rpm, d_0 = 50 mm, V_L = 20 mL, $OD_{600,start}$ = 0.1 [-].

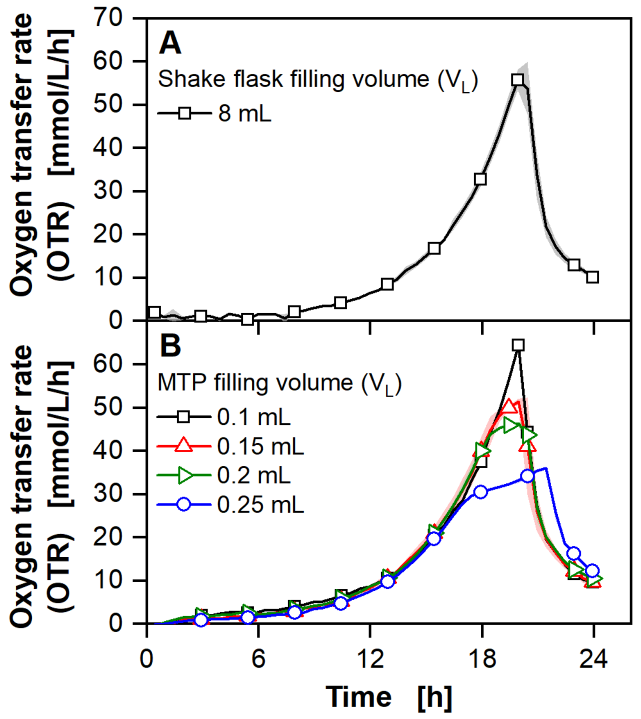

Figure S3: OTR-based scale-down of cultivation conditions from (A) shake flask to (B) MTP scale. For clarity, only every fifth data point overtime is represented as a symbol. Lines are drawn through all measuring points. Shadows indicate the minimum and maximum values of biological duplicates. Cultivation conditions: *U. trichophora*, (**A**) RAMOS device, 250 mL shake flasks, n = 350 rpm, d_0 = 50 mm, V_L = 8 mL, (**B**) µTOM device, 96 round deep well plates, n = 1000 rpm, d_0 = 3 mm, V_L = 0.1-0.25 mL, modified Verduyn medium (25 g/L sucrose, 6 g/L NH_4Cl, 2 g/L KH_2PO_4, 0.4 g/L $MgSO_4$, 0.3 M MES (pH 7.2)), T = 30 °C, $OD_{600,start}$ = 0.1 [-].

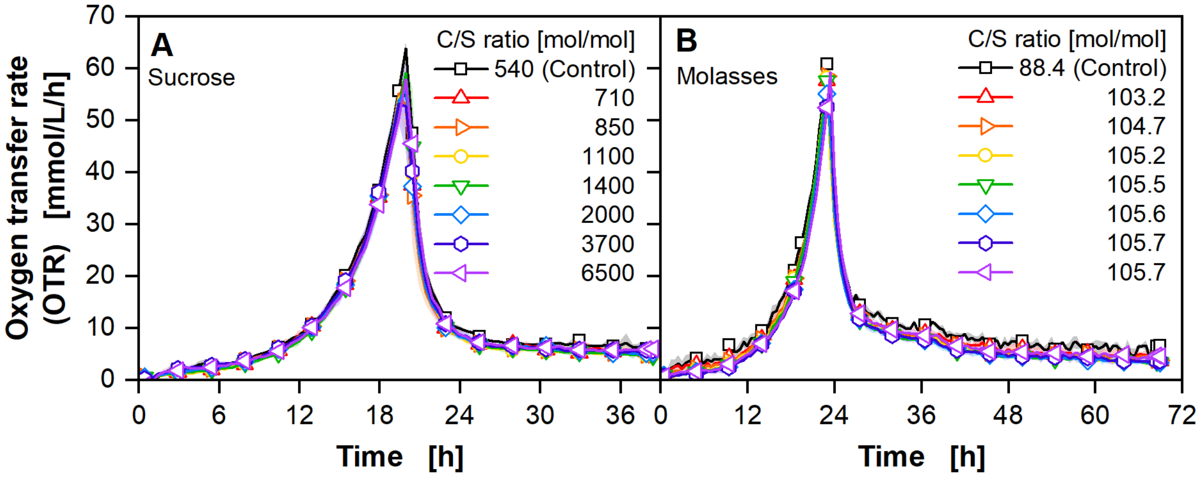

Figure S4: No secondary substrate limitation was observed for the investigated sulphate concentrations. (**A**) Sucrose was used as the carbon source. For clarity, only every fifth data point over time is represented as a symbol. (**B**) Molasses was used as the carbon source. For clarity, only every ninth data point over time is represented as a symbol. (**A**) – (**B**) Lines are drawn through all data points. Shadows indicate standard deviation of biological triplicates. Cultivation conditions: *U. trichophora*, µTOM device, 96 round deep well plates, modified Verduyn medium ((**A**) 25 g/L sucrose, (**B**) 25 g/L sucrose in molasses, 6 g/L NH_4Cl, 2 g/L KH_2PO_4, (**A**) 0.025-0.4 g/L $MgSO_4$, (**B**) 0-0.4 g/L $MgSO_4$, 0-0.33 g/L $MgCl_2 * 6 H_2O$, 0.3 M MES (pH 7.2)), T = 30 °C, n = 1000 rpm, d_0 = 3 mm, V_L = 100 µL, $OD_{600,start}$ = 0.1 [-].

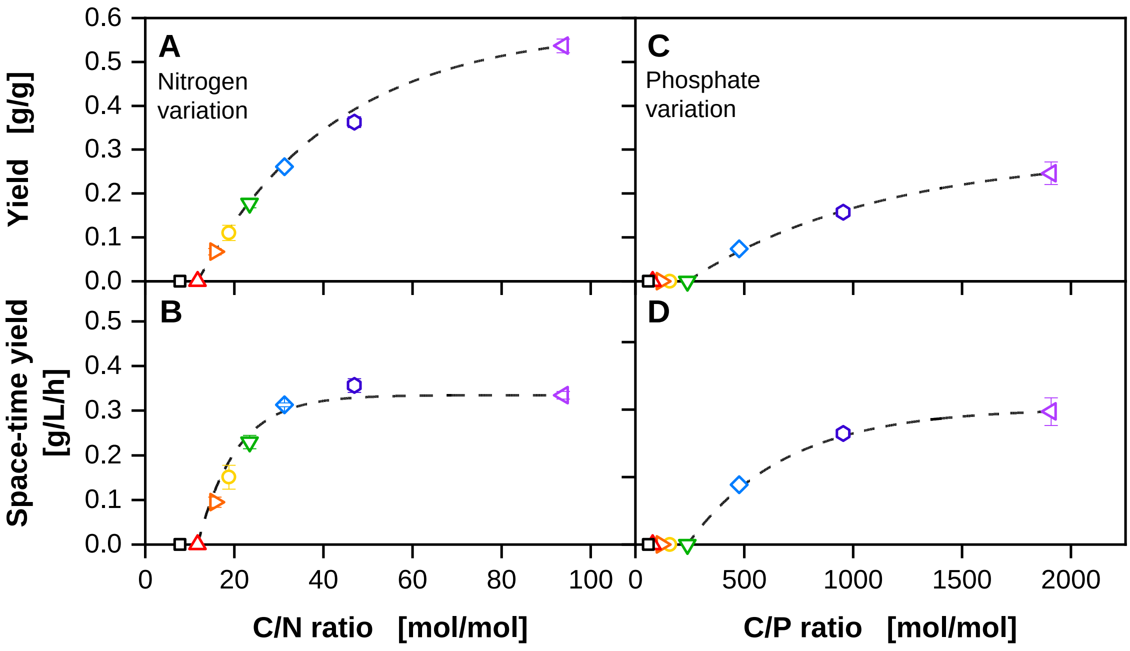

Figure S5: Effect of nitrogen and phosphate limitation on malic acid yield and space-time yield on sucrose. (**A**), (**C**) Malic acid yield. (**B**), (**D**) Space-time yield. Corresponding OTRs and malic acid concentrations are given in Figure 2. Cultivation conditions: *U trichophora*, µTOM device, 96 round deep well plates, medium: modified Verduyn medium (25 g/L sucrose, 0.4 g/L $MgSO_4$, 0.3 M MES (pH 7.2)), T = 30 °C, n = 1000 rpm, d_0 = 3 mm, V_L = 100 µL, $OD_{600,start}$ = 0.1 [-]. (**A**), (**B**) 0.5-6 g/L NH_4Cl, 0-6.6 g/L NaCl, 2 g/L KH_2PO_4. (**C**), (**D**) 6 g/L NH_4Cl, 0.06-2 g/L KH_2PO_4, 0-1.1 g/L KCl. Error bars indicate standard deviation of biological triplicates.

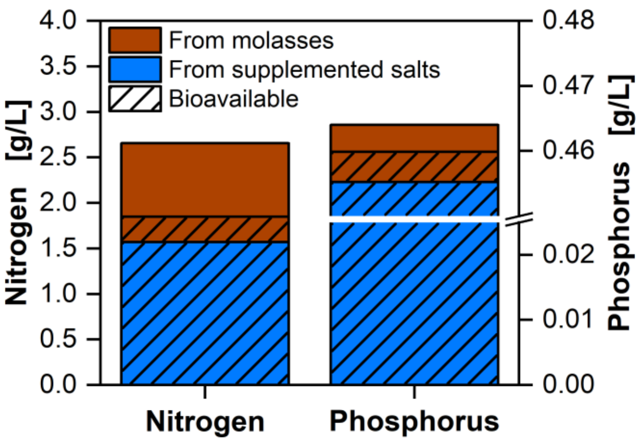

Figure S6: Concentration, origin and bioavailability of nitrogen and phosphorus in adapted Verduyn medium with molasses. Adapted Verduyn medium for unlimited growth used for experiments in Figure 3: molasses equivalent to 25 g/L sucrose, 6 g/L NH_4Cl, 2 g/L KH_2PO_4.

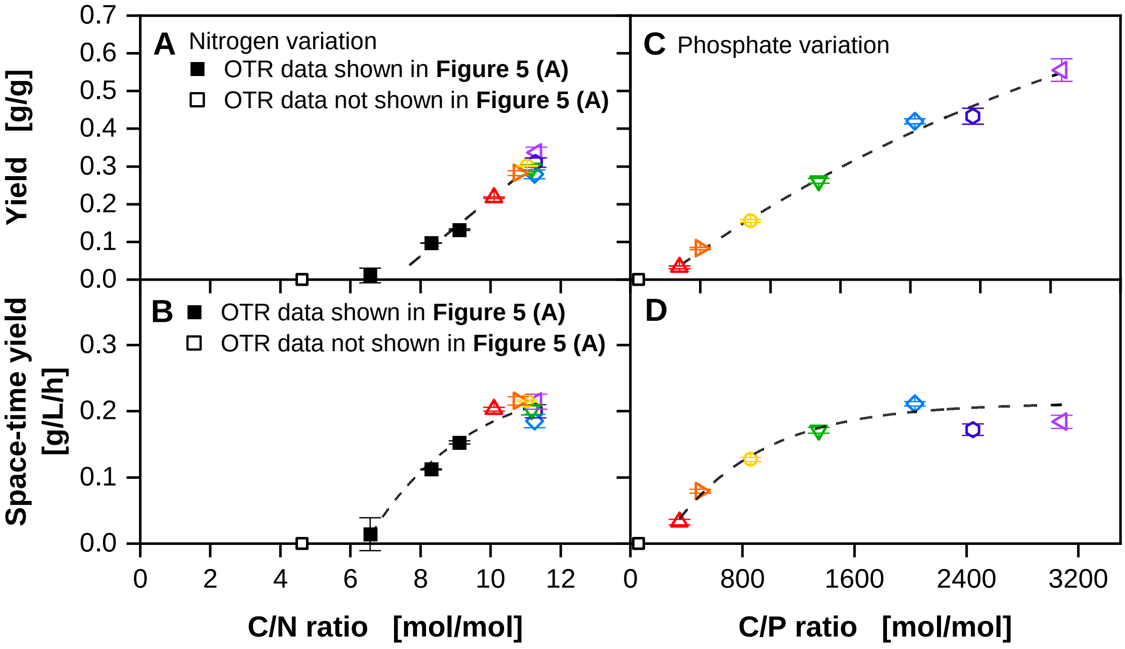

Figure S7: Effect of nitrogen and phosphate limitation on malic acid yield and space-time yield on molasses. (**A**), (**C**) Malic acid yield. (**B**), (**D**) Space-time yield. (**A**), (**B**) Filled symbols indicate samples, which were taken from the experiment shown in Figure 3 (**A**). Open symbols indicate samples, which were taken from an additional experiment, investigating different C/N ratios. Corresponding OTRs and malic acid concentrations are given in Figure 3. Cultivation conditions: *U trichophora*, µTOM device, 96 round deep well plates, modified Verduyn medium (25 g/L sucrose in molasses, 0.4 g/L $MgSO_4$, 0.3 M MES (pH 7.2)), T = 30 °C, n = 1000 rpm, d_0 = 3 mm, V_L = 100 µL, $OD_{600,start}$ = 0.1 [-]. (**A**), (**B**) 0-6 g/L NH_4Cl, 0-6.6 g/L NaCl, 2 g/L KH_2PO_4. (**C**), (**D**) 6 g/L NH_4Cl, 0-2 g/L KH_2PO_4, 0-1.1 g/L KCl. Error bars indicate standard deviation of biological triplicates.

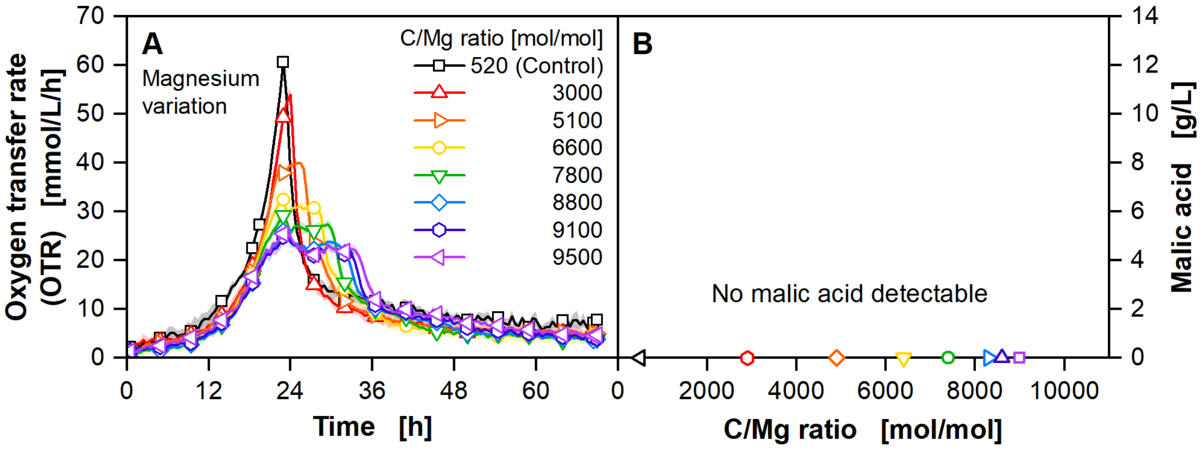

Figure S8: Impact of magnesium limitation on fermentations with the complex substrate molasses. Influence of decreasing magnesium concentrations on (**A**) OTR and (**B**) malic acid production. (**A**) For clarity, only every ninth data point over time is represented as a symbol. Lines are drawn through all measuring points. Shadows indicate standard deviation of biological triplicates. (**B**) Samples were drawn after the sugar was fully consumed, as indicated by a drop in the OTR. Error bars indicate standard deviation of biological triplicates. The respective C/Mg ratios were calculated based on the total amount of magnesium in molasses [18], and the additional salt. Cultivation conditions: *U. trichophora*, µTOM device, 96 round deep well plates, modified Verduyn medium (molasses equivalent to 25 g/L sucrose, 0.3 M MES (pH 7.2), 6 g/L NH_4Cl, 2 g/L KH_2PO_4, 0.025-0.4 g/L $MgSO_4$, 0-0.23 g/L Na_2SO_4), T = 30 °C, n = 1000 rpm, d_0 = 3 mm, V_L = 100 µL, $OD_{600,start}$ = 0.1 [-].

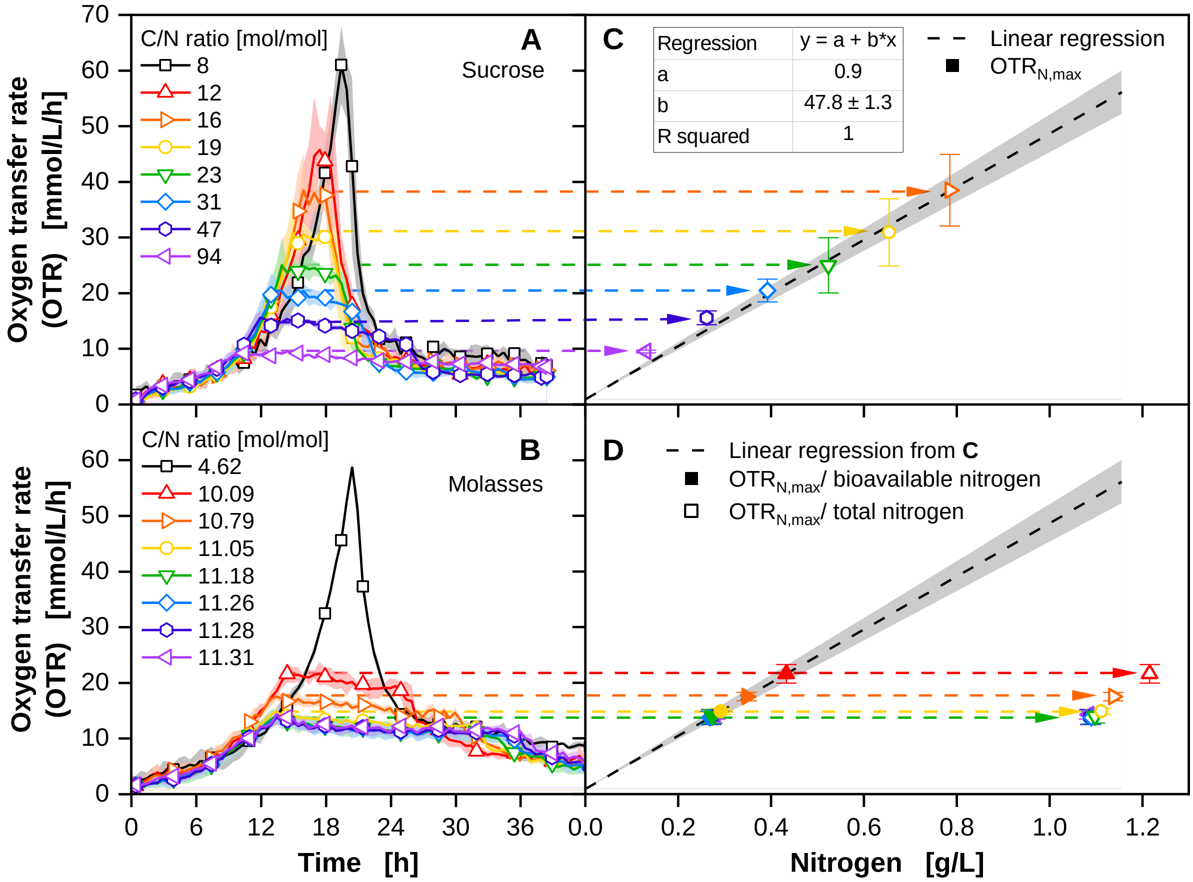

Figure S9: Estimation of biologically available nitrogen in molasses. (**A**), (**C**) Determination of a correlation between the maximum obtained OTR ($OTR_{N,max}$) and supplemented nitrogen concentration from NH_4Cl and (**B**), (**D**) subsequent estimation of biologically available nitrogen in molasses, method according to Niehoff *et al.* [1]. (**A**), (**B**) OTRs of *U. trichophora* (**A**) on defined minimal medium with pure sucrose and (**B**) medium with the complex substrate molasses. For clarity, only (**A**) every fifth and (**B**) seventh data point over time is represented as a symbol. Lines are drawn through all measuring points. Shadows indicate standard deviation of biological triplicates. (**C**) Correlation between $OTR_{N,max}$ and nitrogen concentration of the medium supplemented as NH_4Cl. Only C/N ratios reaching a fully developed plateau were considered for evaluation. Error bars indicate standard deviation of biological triplicates. Shadow indicates 95% confidence band. (**D**) Open symbols indicate that the correlation obtained in (**C**) was used to calculate the nitrogen concentration. Filled symbols indicate that the elemental analysis from Helm *et al.* [2] was used to calculate the nitrogen concentration of the medium supplemented by the molasses and additional NH_4Cl. The cultivation conditions for the data shown in (**A**) and (**B**) are given in Figure 2 and Figure 3, respectively.

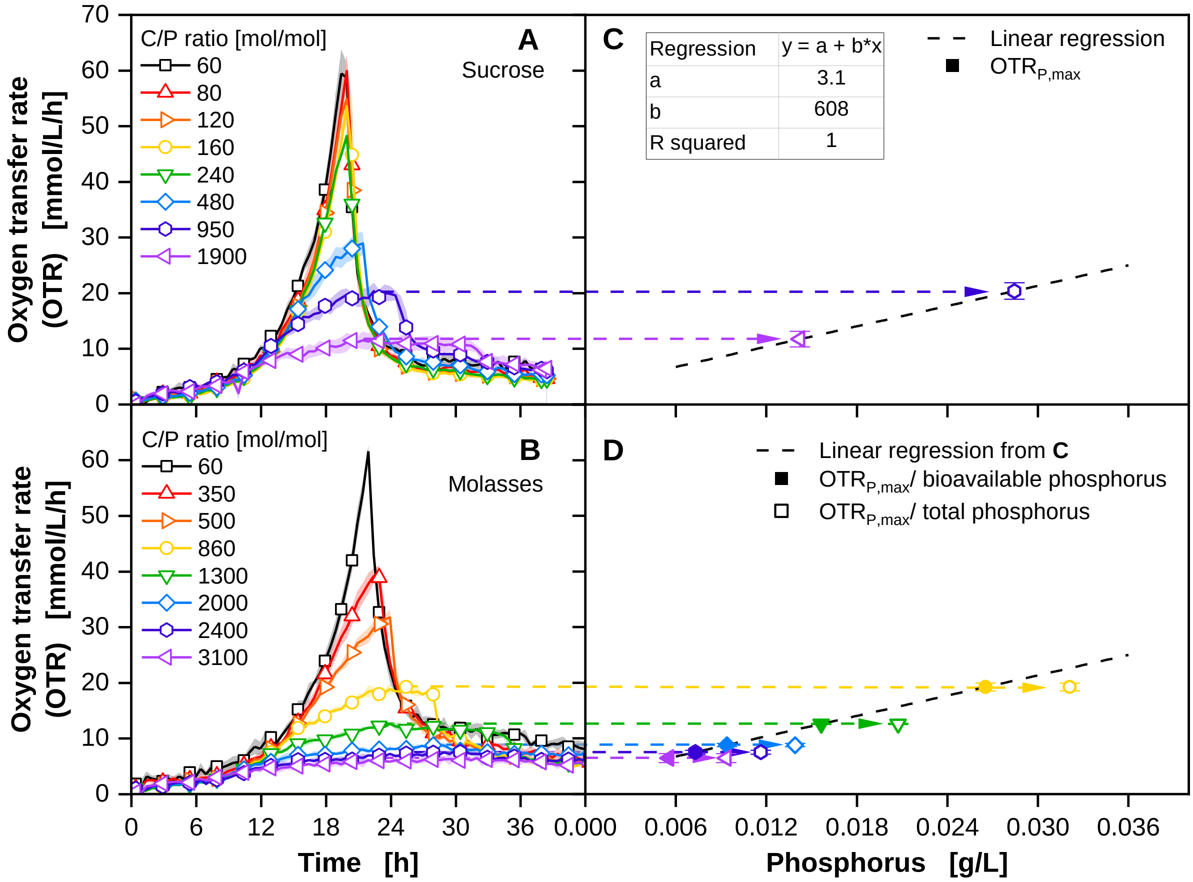

Figure S10: Estimation of biologically available phosphorus in molasses. (**A**), (**C**) Determination of a correlation between $OTR_{P,max}$ and supplemented phosphorus concentration from KH_2PO_4, and (**B**), (**D**) subsequent estimation of biologically available phosphorus in molasses, method according to Niehoff *et al.* [1]. (**A**), (**B**) OTRs of *U. trichophora* on (**A**) defined minimal medium with pure sucrose and (**B**) medium with the complex substrate molasses. For clarity, only every (**A**) fifth and (**B**) seventh data point over time is represented as a symbol. Lines are drawn through all measuring points. Shadows indicate standard deviation of biological triplicates. (**C**) Correlation between $OTR_{P,max}$ and phosphorus concentration of the medium supplemented as KH_2PO_4. Error bars indicate standard deviation of biological triplicates. Only C/P ratios reaching a fully developed plateau were used. (**D**) Open symbols indicate that the correlation obtained in (**C**) was used to calculate the phosphorus concentration. Filled symbols indicate that the elemental analysis from Helm *et al.* [2] was used to calculate the phosphorus concentration of the medium supplemented by the molasses and additional KH_2PO_4. The cultivation conditions for the data shown in (**A**) and (**B**) are given in Figure 2 and Figure 3, respectively.

References

1. Niehoff P-J, Müller W, Pastoors J, Miebach K, Ernst P, Hemmerich J, et al. Development of an itaconic acid production process with Ustilaginaceae on alternative feedstocks. BMC Biotechnol. 2023;23:34. doi:10.1186/s12896-023-00802-9.
2. Helm T, Niehoff P-J, Gätgens J, Stausberg T, Pichler B, Häßler T, et al. Introducing molasses as an alternative feedstock into itaconate production using *Ustilago* sp. N Biotechnol. 2023;77:30–9. doi:10.1016/j.nbt.2023.06.003.
